# Induced regeneration of articular cartilage – identification of a dormant regeneration program for a non-regenerative tissue

**DOI:** 10.1242/dev.201894

**Published:** 2023-11-08

**Authors:** Yu-Lieh Lin, Ling Yu, Mingquan Yan, Katherine Zimmel, Osama Qureshi, Felisha Imholt, Tao Li, Ivan Ivanov, Regina Brunauer, Lindsay Dawson, Ken Muneoka

**Affiliations:** ^1^ Department of Veterinary Physiology and Pharmacology, School of Veterinary Medicine and Biomedical Sciences, Texas A&M University, College Station, TX 77843, USA; ^2^ Department of Hand Surgery, Union Hospital, Tongli Medical College, Huazhong University of Science and Technology, Wuhan, Hubei 430022, People's Republic of China

**Keywords:** Articular cartilage, Regeneration, Fibroblasts, BMP

## Abstract

A mouse organoid culture model was developed to regenerate articular cartilage by sequential treatment with BMP2 and BMP9 (or GDF2) that parallels induced joint regeneration at digit amputation wounds *in vivo*. BMP9-induced chondrogenesis was used to identify clonal cell lines for articular chondrocyte and hypertrophic chondrocyte progenitor cells from digit fibroblasts. A protocol that includes cell aggregation enhanced by BMP2 followed by BMP9-induced chondrogenesis resulted in the differentiation of organized layers of articular chondrocytes, similar to the organization of middle and deep zones of articular cartilage *in situ*, and retained a differentiated phenotype following transplantation. In addition, the differentiation of a non-chondrogenic connective tissue layer containing articular chondrocyte progenitor cells demonstrated that progenitor cell sequestration is coupled with articular cartilage differentiation at a clonal level. The studies identify a dormant endogenous regenerative program for a non-regenerative tissue in which fibroblast-derived progenitor cells can be induced to initiate morphogenetic and differentiative programs that include progenitor cell sequestration. The identification of dormant regenerative programs in non-regenerative tissues such as articular cartilage represents a novel strategy that integrates regeneration biology with regenerative medicine.

## Teaser

**Summary:** The identification of a dormant regenerative program for a non-regenerative tissue that can be extrinsically stimulated provides a translational interface between regeneration biology and regenerative medicine.

## INTRODUCTION

Articular cartilage (AC) is a highly specialized tissue composed of distinct layers of chondrocytes that cover the terminal end of bones, and it prevents skeletal wear and tear by buffering mechanical loading associated with movement (Carballo et al., 2017). As a tissue, AC does not turn over and, when damaged, it lacks regenerative capability, so injury associated with degenerative conditions is characterized by permanent loss of functional tissue. Degenerative diseases of AC affect a large proportion of the population and are a primary cause of physical disabilities (Barbour et al., 2017). For this reason, numerous tissue-engineering strategies targeting AC for surgical replacement have been developed; however, effective clinical outcomes remain limited (Correa and Lietman, 2017; Medvedeva et al., 2018). As AC is non-regenerative *in vivo*, the absence of an endogenous regenerative model that provides strategic guidance for tissue engineering has been lacking. The discovery that joint regeneration *in vivo* can be stimulated at digit amputation wounds by sequential treatment with BMP2 and BMP9 (GDF2) (Yu et al., 2019) established a regeneration model that can bridge approaches in tissue engineering with the biology of regeneration. Induced regeneration studies identify sources of progenitor cells at non-regenerative injuries, such as amputation, that possess the potential for participation in a regeneration response (regenerative potential) (Dolan et al., 2018). Recently, an organoid culture strategy for hyaline cartilage regeneration was developed (Yu et al., 2022) that is distinct from tissue engineering approaches because it relies on the self-organization of dissociated cells, similar to classic models of sponge regeneration (Wilson, 1907). Using this organoid/regeneration protocol, digit-derived fibroblasts from the terminal phalangeal element (P3 cells) (Wu et al., 2013) were identified as a source of chondroprogenitor cells to regenerate hyaline cartilage (Yu et al., 2022). P3 cell lines were established from primary cultures and displayed position-specific characteristics *in vitro* while retaining regenerative potential following transplantation *in vivo* (Wu et al., 2013). This hyaline cartilage regeneration model represents a way to begin a mechanistic exploration of how to regenerate AC.

AC has a complex organization, consisting of multiple layers of chondrocytes, each with specific functions and distinct morphologies characterized by cell size, cell density and extracellular matrix (ECM) composition (Di Bella et al., 2015). During development, hyaline cartilage initially forms by condensation and gives rise to AC of joints in addition to growth plate (GP) cartilage. AC of the knee has a layered organization and is divided into four zones: superficial, middle, deep and calcified. Chondrocytes of the superficial zone are small, flattened cells containing relatively low proteoglycan levels and are identified based on proteoglycan 4 (*Prg4*, also called lubricin) expression (Saito, 2022). Developmental cell lineage studies based on *Prg4* expression show that superficial cells contribute to all zones of AC indicative of progenitor characteristics (Decker et al., 2017; Kozhemyakina et al., 2015; Li et al., 2017). The middle and deep zones contain spheroid chondrocytes, described as hyaline or articular, expressing high levels of collagen II (ColII) and aggrecan (Acan), and are distinguished based on chondrocyte size and density; chondrocytes are smaller and more densely packed in the middle zone than in the deep zone (Becerra et al., 2010; Sophia Fox et al., 2009). The calcified zone contains large chondrocytes identified as hypertrophic based on expression of collagen X (*Col10a1*), runt-related transcription factor 2 (*Runx2*) and matrix metalloproteinase 13 (*Mmp13*) (Chau et al., 2014), and separates the upper zones of articular chondrocytes from the subchondral bone. The tidemark that separates the deep zone from the calcified zone identifies the non-calcified/calcified cartilage interface and is histologically distinct (Havelka et al., 1984).

In this study, the chondrogenic response of mouse P3 fibroblasts was explored by generating clonal cell lines and characterizing responsiveness to BMP9. When cultured under identical conditions, some clonal lines differentiated into articular chondrocytes, whereas others differentiated into hypertrophic chondrocytes. A genome-wide RNA-sequencing (RNAseq) analysis determined that BMP9 directs both articular and hypertrophic clonal lines on a trajectory toward differentiation of AC and not GP cartilage. The cartilage differentiation protocol was refined to include BMP2-enhanced aggregation of suspended cells prior to BMP9-stimulated differentiation. Cartilage differentiated by an articular chondrocyte-specific progenitor line formed a composite tissue with articular chondrocytes encapsulated by a non-chondrogenic fibrous connective tissue layer. The articular chondrocytes were organized into concentric layers and displayed an organization similar to the middle and deep zones of AC *in situ*, and this differentiated phenotype was stable following *in vivo* transplantation. The encapsulating connective tissue layer contained articular chondrocyte-specific progenitor cells, indicating that induced AC differentiation anticipates future injury by sequestering progenitor cells. This *in vitro* model identifies several key transitional steps that include progenitor cell activation, cell expansion, morphogenesis, differentiation and progenitor cell sequestration, thus identifying an endogenous but dormant regenerative program for AC. The identification of dormant regenerative programs in non-regenerative tissues such as AC represents a novel strategy for integrating regeneration biology with regenerative medicine to target solutions for the clinical problem of joint-related disabilities.

## RESULTS

Previously, hyaline cartilage regenerated from BMP9-treated P3 fibroblasts *in vitro* failed to differentiate into AC after transplantation *in vivo* (Yu et al., 2022). Joint repair *in vivo* thus requires a need to develop strategies to further differentiate AC *in vitro* prior to implantation. To aid such studies, the neonatal knee (postnatal day 7) served as an example of maturing AC as the upper layers of articular chondrocytes were distinct at this stage (Fig. S1A,B). Safranin O/Fast Green staining identifies a matrix rich in glycosaminoglycans that is known to be abundant in the AC matrix (Schmitz et al., 2010), and Mallory's trichrome staining identifies collagen fibers based on Aniline Blue staining (Yu et al., 2022). The maturing AC is characterized by a gradient of articular chondrocyte size, with small chondrocytes associated with the superficial zone and progressively larger chondrocytes in the middle and deep zones. At this developmental stage, differentiation into a distinct calcified zone has not occurred. Articular chondrocytes of the neonatal knee were immature, and most cells expressed Sox9 (Fig. S1C). Superficial zone chondrocytes were identified based on the expression of Prg4 (Fig. S1D). The middle zone contained chondrocytes of intermediate size and were characterized by expression of Cilp, a matrix component with phosphatase activity (Bernardo et al., 2011; Lorenzo et al., 1998), and reduced expression of Acan (Fig. S1E,F). We noted that some superficial zone chondrocytes were positive for Cilp. Chondrocytes in the deep zone were larger with prominent lacunae and robust Acan expression (Fig. S1F). The neonatal AC abutted chondrocytes of the GP that were organized with resting chondrocytes, giving rise to columns of proliferating chondrocytes that differentiated into collagen X (ColX)-expressing hypertrophic chondrocytes (Fig. S1G). The developing epiphysis was surrounded by a fibrous stromal tissue layer characterized by collagen I (ColI) expression (Fig. S1H).

### Cloning of chondrocyte-specific progenitor cell lines

**Fig. 1.**
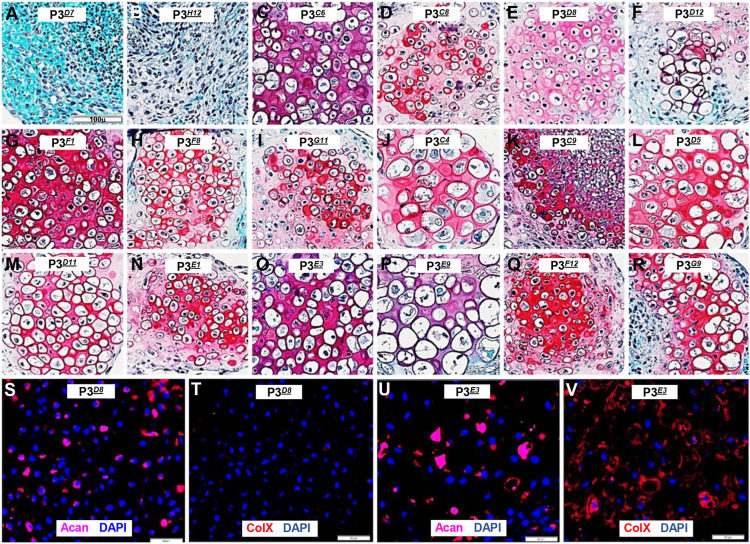
**BMP9-induced chondrogenesis of P3 clonal cell lines.** Pellet cultures of 18 clonal lines were cultured in BMP9 differentiation medium and analyzed histologically based on Safranin O/Fast Green staining (*n*=2). (A,B) Two clones failed to differentiate into histologically identifiable chondrocytes. (C-R) 16 clones differentiated into chondrocytes containing a matrix rich in glycosaminoglycan. Each clone differentiated into chondrocytes of uniform size, but chondrocyte size varied between clones. (S,T) Immunohistochemical staining of P3*^D8^* (E) cartilage identified cells positive for Acan (S) and negative for ColX (T). (U,V) Immunohistochemical staining of P3*^E3^* (O) cartilage identified cells positive for Acan (U) and positive for ColX (V). Scale bars: 100 µm (A, applies to B-R); 50 µm (S-V).

There are two crucial variables for generating cartilage in culture: cell source and differentiation protocol. With respect to the cell source, P3 fibroblasts differentiate into both articular and hypertrophic chondrocytes following treatment with BMP9 (Yu et al., 2022), so clonal P3 lines were established to determine whether distinct chondroprogenitor cells could be isolated. *lacZ^+^* P3 cells (Wu et al., 2013) were cloned by dilution and assayed for chondrogenic potential based on their response to BMP9. A total of 29 clonal lines were isolated from a single 96-well plate (30.2% efficiency), expanded and cryopreserved. To determine chondrogenic potential, 18 clonal lines were cultured as centrifuged cell pellets (*n*=2), treated with BMP9 differentiation medium for 21 days, processed for histology and analyzed based on staining with Safranin O/Fast Green. Chondrocytes were identified based on cell morphology (spherical cells with lacuna) and Safranin O-positive matrix staining. Two clones (2/18, 11%) were negative for Safranin O staining (Fig. 1A,B), whereas 16 clones (16/18, 89%) were positive (Fig. 1C-R). Each clone differentiated into chondrocytes that were uniform in size, but chondrocyte size varied between the 16 clones. The clones containing smaller chondrocytes were tentatively identified as articular chondroprogenitors, whereas the clones containing larger chondrocytes were identified as hypertrophic chondroprogenitors. To explore this, the phenotype of two chondroprogenitor clonal lines representing small (P3*^D8^*; Fig. 1G) and large (P3*^E3^*; Fig. 1L) chondrocytes were analyzed by immunohistochemical staining for Acan and ColX. P3*^D8^* clonal cells differentiated into small chondrocytes that were positive for Acan but negative for ColX, consistent with an articular chondrocyte phenotype (Fig. 1S,T). P3*^E3^* clonal cells differentiated into large chondrocytes that were positive for both Acan and ColX, consistent with a hypertrophic chondrocyte phenotype (Fig. 1U,V). We further analyzed the remaining 14 clones and found six additional clones that were positive for Acan and negative for ColX (Fig. 1C-I; P3*^C6^*, P3*^C8^*, P3*^D12^*, P3*^F1^*, P3*^F8^* and P3*^G11^*), and eight additional clones positive for both Acan and ColX (Fig. 1J-R; P3*^C4^*, P3*^C9^*, P3*^D5^*, P3*^D11^*, P3*^E1^*, P3*^E9^*, P3*^F12^* and P3*^G9^*). Together, the histological and immunohistochemical studies support the conclusion that the P3 cell line contains chondrocyte-specific progenitor cells that displayed distinct responses to BMP9.

### Optimizing culture conditions for efficient cartilage differentiation

**Fig. 2.**
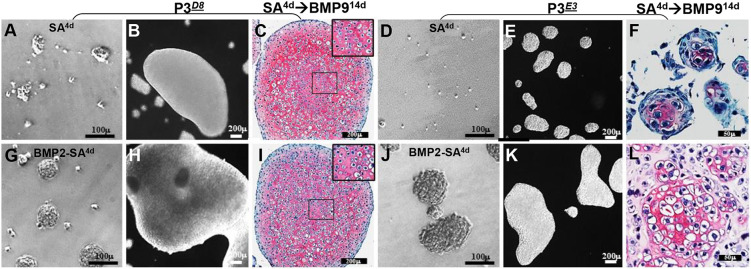
**BMP2-enhanced cell aggregation of P3*^D8^* and P3*^E3^* clonal cells.** (A) After 4 days in suspension culture, P3*^D8^* cells form aggregates of varying size. (B) After 14 days in BMP9 differentiation medium, P3*^D8^* aggregates form small and large cell clusters. (C) Cross-section of a large P3*^D8^* cluster stained with Safranin O/Fast Green showing cartilage tissue densely populated with chondrocytes embedded in a matrix rich in glycosaminoglycans (*n*=3). The inset shows central chondrocytes at a higher magnification. (D) After 4 days in suspension culture, P3*^E3^* cells form very small aggregates. (E) After 14 days in BMP9 differentiation medium, P3*^E3^* aggregates form small cell clusters. (F) Safranin O/Fast Green staining of P3*^E3^* clusters identifies chondrocytes but with low glycosaminoglycan production (*n*=3). (G) After 4 days in suspension culture with BMP2, P3*^D8^* cells form large aggregates. (H) After 14 days in BMP9 differentiation medium, P3*^D8^* aggregates form large cell clusters. (I) Safranin O/Fast Green staining of P3*^D8^* clusters identifies cartilage densely populated with chondrocytes embedded in a matrix rich in glycosaminoglycans (*n*=3). The inset shows central chondrocytes at a higher magnification. (J) After 4 days in suspension culture with BMP2, P3*^E3^* cells form large aggregates. (K) After 14 days in BMP9 differentiation medium, P3*^E3^* aggregates form large cell clusters. (L) Safranin O/Fast Green staining of P3*^E3^* clusters identifies aggregates of large chondrocytes embedded in Safranin O staining matrix (*n*=3). Scale bars: 100 µm (A,D,G,J); 200 µm (B,C,E,H,I,K); 50 µm (F,L).

The P3*^D8^* and P3*^E3^* clonal lines were selected as candidate chondroprogenitor lines for detailed studies. We first explored the effect of culture condition for cartilage differentiation. Previous studies of the parental P3 line established a self-aggregation (SA) protocol of 4 days in basal medium followed by treatment with BMP9 differentiation medium (SA^4d^→BMP9) to regenerate hyaline cartilage (Yu et al., 2022). When P3*^D8^* clonal fibroblasts were cultured using the SA protocol, they displayed a variable aggregation response, forming many small aggregates and a few large aggregates (Fig. 2A). After 14 days in BMP9 differentiation medium (SA^4d^→BMP9^14d^), the large aggregates formed large cell clusters, whereas the small aggregates formed small cell clusters (Fig. 2B). Histological analysis revealed chondrocytes in both small and large clusters, but only cells of large clusters differentiated into chondrocytes embedded in a glycosaminoglycan rich matrix comparable with that of AC (Fig. 2C). Thus, the SA^4d^→BMP9^14d^ protocol was effective in regenerating articular chondrocytes from P3*^D8^* clonal cells (Fig. 2C, inset); however, the response efficiency was limited by the initial aggregation response. Alternatively, parallel studies with P3*^E3^* clonal cells indicated a poor SA response (Fig. 2D), but after treatment in BMP9 differentiation medium (SA^4d^→BMP9^14d^), chondrocytes were identified in the resulting small cell clusters (Fig. 2E,F). Thus, P3 clonal lines retain chondrogenic potential in BMP9-treated SA cultures, but this response is limited by the extent of SA.

To improve the SA protocol, we explored ways to enhance cell aggregation. During BMP9-induced joint regeneration, hyaline cartilage condenses and the regeneration response is enhanced by pre-treatment with BMP2 (Yu et al., 2019). During cartilage development, condensation of hyaline cartilage is initiated by various growth factors, including BMP2 (Giffin et al., 2019). To determine whether BMP2 pre-treatment improved the SA response *in vitro*, suspension cultures of P3*^D8^* and P3*^E3^* clonal lines were treated with BMP2 aggregation medium during the 4-day SA period. BMP2 treatment (BMP2-SA^4d^) resulted in the formation of large aggregates in both P3*^D8^* and P3*^E3^* clonal lines (Fig. 2G,J). After subsequent treatment in BMP9 differentiation medium (BMP9^14d^), both clonal lines formed numerous large cell clusters (Fig. 2H,K) and, when assayed histologically, the BMP2-SA^4d^→BMP9^14d^ treatment protocol regenerated cartilage rich in glycosaminoglycans; P3*^D8^* clonal cells differentiated into cartilage with small chondrocytes (Fig. 2I, inset), whereas P3*^E3^* clonal cells differentiated into cartilage containing large chondrocytes (Fig. 2I,L). These results indicate that the chondrogenic response was enhanced by BMP2 treatment during the SA period and this protocol (BMP2-SA^4d^→BMP9) was adopted for further regeneration studies.

### Comparative RNAseq analyses suggest that the P3*^D8^* and P3*^E3^* clonal lines are progenitors for AC

**Fig. 3.**
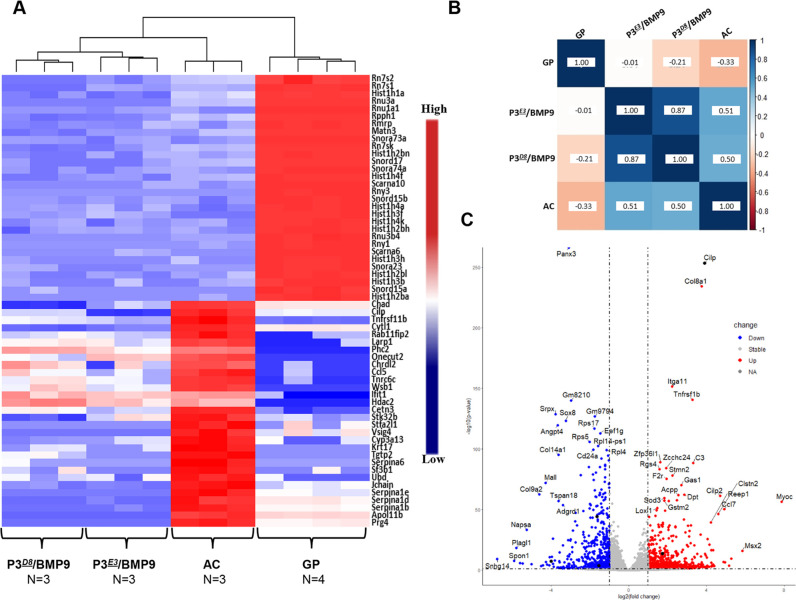
**Genome-wide RNAseq analysis of P3*^D8^* and P3*^E3^* cartilage.** P3*^D8^* and P3*^E3^* clonal cells were differentiated using the BMP2-SA^4d^→BMP9^14d^ protocol and labeled as P3*^D8^*/BMP9 and P3*^E3^*/BMP9, respectively. RNAseq datasets were generated from three separate experiments for each cell line. (A) Heat map and clustering analysis of the selected set of 60 DE genes identified by a genome-wide analysis of genes expressed by articular cartilage (AC) (*n*=3) and growth plate (GP) cartilage (*n*=4). (B) Correlational analysis based on the selected set of 60 DE genes. (C) Volcano plot summarizing the DE genes resulting from the comparison of P3*^D8^*/BMP9- versus P3*^E3^*/BMP9-derived cartilage, with 50 outlying genes labeled based on lowest adjusted *P*-value and highest log_2_(fold change) identified. Down, downregulated; Up, upregulated; NA, not applicable.

The differentiative trajectory of P3*^D8^* and P3*^E3^* cells induced by the BMP2-SA^4d^→BMP9^14d^ protocol was explored using RNAseq transcriptome analyses of datasets (*n*=3) generated from *in vitro*-differentiated cartilage (Gene Expression Omnibus accession number GSE216025). To probe the specific chondrogenic responses of P3*^D8^*- and P3*^E3^*-derived cartilage, the datasets were compared with RNAseq datasets of murine AC (Bekki et al., 2020) and murine GP cartilage (Sabik et al., 2017). To distinguish AC from GP cartilage, a correlation analysis of all expressed genes indicated a positive correlation (r=+0.78), which concurs with their similar developmental origin and indicates that the two cartilage tissues are not easily distinguishable by this method. A genome-wide comparison of AC and GP cartilage transcriptomes identified 5142 differentially expressed (DE) genes [log_2_(fold change or FC)≥|1.5| and false discovery rate (FDR) *P*-value<0.05], of which 2369 were upregulated in GP cartilage and 2439 were upregulated in AC (Table S1). A complementary correlation analysis restricted to DE genes also detected a positive correlation (r=+0.65) (Fig. S2) between AC and GP cartilage. To distinguish AC from GP cartilage, the top 30 DE genes from each list were used for a correlation analysis and the combined set of 60 DE genes detected a negative correlation (r=−0.33). Thus, this set of 60 genes was used for a comparative evaluation of the chondrogenic responses of P3*^D8^* and P3*^E3^* cells. A comparative heat map with normalized gene expression values, including a clustering analysis of AC, GP cartilage and cartilage differentiated from P3*^D8^* and P3*^E3^* cells, is shown in Fig. 3A, and an overall correlation analysis of the same set of data using the average gene expression values is shown in Fig. 3B. Whereas AC and GP cartilage were negatively correlated (r=−0.33), the cartilage differentiated from P3*^D8^* cells displayed a negative correlation compared with GP cartilage (r=−0.21) and a positive correlation compared with AC (r=+0.50). This supports the conclusion that differentiated P3*^D8^* clonal cells display a bias toward AC compared with GP cartilage. Similarly, the cartilage differentiated from P3*^E3^* cells displayed a neutral relationship compared with GP cartilage (r=−0.01) and a positive correlation compared with AC (r=+0.51), suggesting that hypertrophic chondrocytes differentiated from P3*^E3^* cells are more similar to AC than to GP cartilage (Fig. 3A,B). These findings suggest that P3*^E3^*-derived chondrocytes were differentiating into the hypertrophic chondrocytes of the AC, i.e. chondrocytes of the calcified layer (Carballo et al., 2017), and are distinct from the hypertrophic chondrocytes of the GP. A positive correlation of r=+0.50 does not support the conclusion that P3*^D8^*- or P3*^E^*-derived cartilage is identical to AC; however, a complicating factor is that AC is composed of both articular and hypertrophic chondrocytes, whereas the cartilage differentiated from P3*^D8^* or P3*^E3^* cells is either articular or hypertrophic. Our analysis does support the conclusion that both P3*^D8^* and P3*^E3^* clonal cells treated with the BMP2-SA^4d^→BMP9^14d^ protocol display a differentiation trajectory toward AC.

Consistent with this conclusion, a comparative analysis based on the 60 DE target genes indicated that the cartilage differentiated from P3*^E3^* cells correlated positively (r=+0.87) with cartilage differentiated from P3*^D8^* cells (Fig. 3B). A direct comparison between the complete P3*^E3^* and P3*^D8^* cartilage datasets identified 717 DE transcripts (log_2_FC≥|1.5| and FDR *P*-value<0.05) that included 462 DE genes upregulated in P3*^D8^* cartilage and 255 DE genes upregulated in P3*^E3^* cartilage (Fig. 3C; Table S2). Screening the DE genes with a cartilage-specific gene list (Yu et al., 2022) identified 13 upregulated genes in P3*^E3^*-derived cartilage (*Mmp13*, *Ror2*, *Bmp6*, *Frzb*, *Nog*, *Col11a1*, *Sost*, *Gdf5*, *Epyc*, *Clec3a*, *Ihh*, *Mia* and *Col9a2*) and 16 upregulated genes in P3*^D8^*-derived cartilage (*Msx2*, *Cilp*, *Scx*, *Bmp1*, *Prrx1*, *Egfr*, *Ereg*, *Bmp2*, *Adamts12*, *Dcn*, *Osr1*, *Smad3*, *Enpp2*, *Trps1*, *Itgb8* and *Sfrp2*). Notably, three genes differentially upregulated in P3*^E3^*-derived cartilage (*Ihh*, *Mmp13* and *Ror2*) are known to be specific for hypertrophic chondrocytes (Rim et al., 2020; St-Jacques et al., 1999; Thorup et al., 2020), which supports the conclusion based on histological and immunohistochemical studies that P3*^E3^*-derived cartilage is hypertrophic. Similarly, four upregulated genes in P3*^D8^*-derived cartilage are associated with AC/joint formation (*Cilp*, *Osr1*, *Sfrp2* and *Enpp2*), which supports the conclusion that P3*^D8^*-derived cartilage is articular (Gao et al., 2011; Leimeister et al., 1998; Lorenzo et al., 1998; Ozpolat et al., 2012).

Screening the datasets of DE genes with a database of known transcription factors (Lambert et al., 2018) identified candidates associated with the differentiation of these two subpopulations of chondrocytes. The dataset of genes upregulated in P3*^E3^*-derived cartilage identified eight known transcription factors (*Plagl1*, *Vax2*, *Nfe2l3*, *Sox8*, *Alx3*, *Atf3*, *Batf3* and *Foxd1*), whereas the dataset of P3*^D8^*-derived cartilage identified 32 transcription factors (*Msx2*, *Cxxc4*, *Dmrta1*, *Sox21*, *Rbpj*, *Nrf1*, *Hsf2*, *Scx*, *Zic2*, *Pogk*, *Mbd4*, *Mnt*, *Prrx1*, *Bcl6*, *Zbed6*, *Rel*, *Foxs1*, *Zkscan3*, *Osr1*, *Smad3*, *Vezf1*, *Zbtb5*, *Mafb*, *Bcl6b*, *Setbp1*, *Foxf1*, *Hoxd10*, *Trps1*, *Kdm2a*, *Stat6*, *Jun* and *Bhlhe22*)*.* Notable among the P3*^D8^*-associated genes are genes expressed during limb development (*Msx2*, *Prrx1*, *Osr1* and *Hoxd10*), genes linked to TGFβ (*Smad3*) and Notch (*Rbpj*) signaling, and genes known to be involved in the regeneration of other organ systems, e.g. lung (*Foxf1*) (Bolte et al., 2017) and heart (*Nrf1*) (Cui et al., 2021). To identify global genetic changes, we carried out a KEGG pathway enrichment analysis using the DAVID Functional Annotation Tool (https://david-d.ncifcrf.gov/home.jsp) on genes differentially expressed by P3*^E3^*- and P3*^D8^*-derived cartilage. No pathways were identified in the P3*^E3^*-derived cartilage dataset and two pathways (focal adhesion and PI3K-Akt signaling) were identified in the P3*^D8^* dataset. The focal adhesion pathway is related to mechanotransduction between cells and the ECM and there is considerable evidence that physical force influences cartilage regeneration (Huang et al., 2018). The PI3K-Akt signaling pathway has been linked to chondrocyte differentiation of stem cells (Klampfleuthner et al., 2022) and is also implicated in cartilage degeneration associated with osteoarthritis (Sun et al., 2020).

### Organized AC differentiates from P3*^D8^* clonal cell cultures

**Fig. 4.**
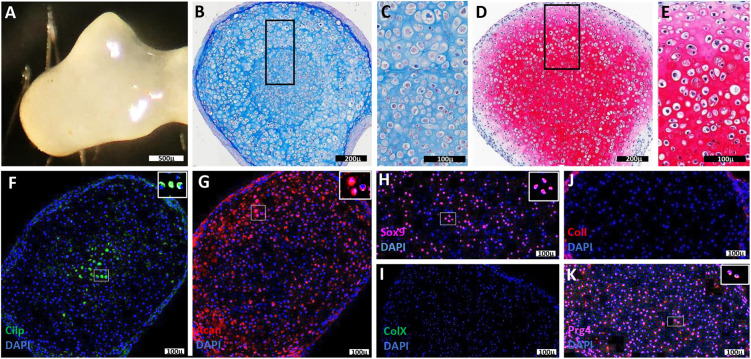
**Maturation of P3*^D8^*-regenerated articular cartilage.** (A) External appearance of cartilage regenerated from P3*^D8^* cells by the BMP2-SA^4d^→BMP9^40d^ protocol (*n*=3). (B) Malory's trichrome staining of P3*^D8^*-regenerated cartilage showing small chondrocytes organized centrally surrounded by larger chondrocytes (*n*=3). (C) Higher magnification of the box indicated in B showing the transition from small to large chondrocytes. (D) Safranin O/Fast Green staining of P3*^D8^*-regenerated cartilage showing differential production of the glycosaminoglycan-rich matrix that is high centrally and low peripherally (*n*=3). (E) Higher magnification of the box indicated in D showing the transition in Safranin O/Fast Green staining. (F) Immunohistochemical staining for Cilp showing positive cells (inset) localized to the central region of the regenerated cartilage. (G) Immunohistochemical staining for Acan showing positive cells (inset) localized to peripheral regions of the regenerated cartilage. (H) Immunohistochemical staining for Sox9 showing positive cells (inset) scattered throughout the regenerated cartilage. (I) Immunohistochemical staining for ColX showing the absence of positive cells in the regenerated cartilage. (J) Immunohistochemical staining for ColI showing the absence of positive cells in the regenerated cartilage. (K) Immunohistochemical staining for Prg4 showing positive cells (inset) scattered throughout the regenerated cartilage. All antibody studies: *n*=3. Scale bars: 500 µm (A); 200 µm (B,D); 100 µm (C,E-K).

For P3*^D8^* clonal cells, cartilage maturation in cultures was assessed using the BMP2-SA^4d^→BMP9 protocol for a total culture period of 44 days (BMP2-SA^4d^→BMP9^40d^). The resulting tissue displayed distinct external cartilage characteristics that included a firm structure and a glossy blue-white appearance (Fig. 4A). When analyzed histologically (*n*=6), the tissue was clearly cartilaginous and displayed a distinct organization that was evident in Mallory's trichrome- and Safranin O/Fast Green-stained sections (Fig. 4B,D). Compared with the 14-day samples, the size of the regenerated cartilage and the general organization of chondrocytes was similar (compare Fig. 4D with Fig. 2I), although the 14-day samples appeared to be immature. Mallory's trichrome staining identified a central core of small chondrocytes that transitioned to larger peripheral chondrocytes (Fig. 4B,C), whereas Safranin O/Fast Green staining identified a gradient of glycosaminoglycan production that was high centrally and low peripherally (Fig. 4D,E). An analysis of adjacent sections stained for Safranin O/Fast Green and Mallory's trichrome identified a concentric organization with small chondrocytes/high glycosaminoglycan production in the center, surrounded by a ring of larger chondrocytes/high glycosaminoglycan production, and a peripheral ring of large chondrocytes/lower glycosaminoglycan production. Thus, the differentiated P3*^D8^* cartilage was organized into concentric layers of chondrocytes displaying a gradient of increasing chondrocyte size and glycosaminoglycan production. This organization was reminiscent of the middle and deep zone cell layers of AC (Fig. S1A), where the smaller chondrocytes of the middle zone graded into the larger chondrocytes of the deep zone. In the case of *in vitro*-regenerated cartilage, the smaller central chondrocytes appeared similar to those of the middle zone and graded into the larger peripheral chondrocytes that were similar to those of the deep zone. Immunostaining studies also supported this conclusion (*n*=3). Cilp-immunopositive cells were abundant in the central core of regenerated chondrocytes, corresponding to middle zone chondrocytes *in vivo*, and Acan-immunopositive cells were the most abundant in the larger peripheral chondrocytes, which correspond to deep zone chondrocytes *in vivo*. (Fig. 4F,G; Fig. S3A-C). Sox9-expressing cells were scattered throughout the regenerated cartilage (Fig. 4H), although not all chondrocytes were immunopositive as they were *in vivo* (Fig. S1C). ColX- and ColI-immunopositive cells were absent in the regenerated cartilage (Fig. 4I,J). The similarity to *in situ* AC did not involve cells of the superficial zone as Prg4-expressing cells were found scattered throughout the cartilage (Fig. 4K; Fig. S3D), and the most peripheral layer of regenerated tissue was non-chondrogenic (see below). Overall, the histological and immunohistochemical analyses suggested that P3*^D8^* clonal cells are induced to regenerate articular chondrocytes of the top three AC zones with the Cilp^+^-middle- and Acan^+^-deep-zone chondrocytes displaying a concentric layered organization. These differentiation studies identify P3*^D8^* cells as a clonal line of articular chondrocyte progenitor cells.

### AC regeneration includes a program to restore regenerative potential

**Fig. 5.**
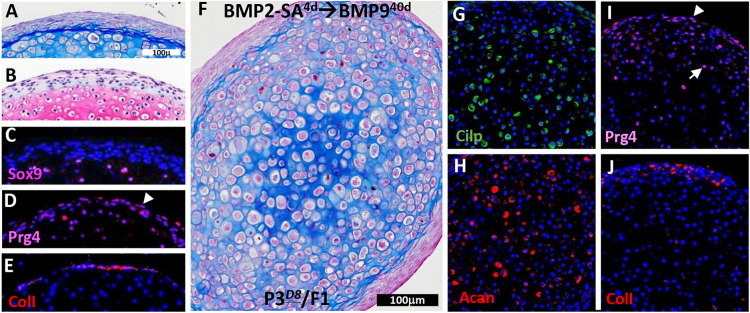
**The encapsulating fibrous tissue layer contains articular cartilage progenitor cells.** (A,B) High-magnification image of the fibrous tissue layer surrounding regenerated cartilage from P3*^D8^* cells by the BMP2-SA^4d^→BMP9^40d^ protocol shown in Fig. 4B,D identified by Mallory's trichrome (A) and Safranin O/Fast Green staining (B) (*n*>6). (C-E) The cells of the fibrous layer were negative for Sox9 (C) and positive for Prg4 (D, arrowhead) and Col1 (E). (F) When cells from the differentiated fibrous layer were isolated (P3*^D8^*/F1) and tested for cartilage regeneration using the BMP2-SA^4d^→BMP9^40d^ protocol, a response similar to P3*^D8^* cells was observed (*n*=4). (G,H) P3*^D8^*/F1-regenerated cartilage contained cells immunopositive for Cilp (G) and Acan (H) expression. (I,J) P3*^D8^*/F1-regenerated tissue contained cells immunopositive for Prg4 (I) in the regenerated cartilage (arrow) and fibrous layer (arrowhead), and ColI (J) was expressed in the fibrous layer. All antibody studies: *n*=3. Scale bars: 100 µm (A, applies for B-E,G-J); 100 µm (F).

P3*^D8^* cells represent an articular chondroprogenitor cell line based on their ability to differentiate into articular chondrocytes by the BMP2-SA^4d^→BMP9^40d^ protocol. Interestingly, although some P3*^D8^* cells differentiated into articular chondrocytes, the cells on the periphery differentiated into a non-chondrogenic fibrous tissue layer (*n*>6) that completely encapsulated the cartilage (Fig. 5A,B). Cells in this layer were positive for ColI (Fig. 5E) and negative for Sox9 (Fig. 5C), although some cells were also positive for Prg4 (Fig. 5D). As the P3*^D8^* line is clonally derived, it is interesting that these cells respond to chondrogenic differentiation medium (basal medium+BMP9) by forming two distinct tissue types; thus, the P3*^D8^*-regenerated cartilage is a clonally derived composite tissue.

The fibrous layer is histologically distinct from the underlying cartilage and similar to the stromal tissue layer associated with the neonatal knee (Fig. S1B) known to contain chondrogenic progenitor cells (Tong et al., 2019). To determine whether articular chondrocyte progenitor cells are present in this fibrous layer, BMP9-regenerated AC (BMP2-SA^4d^→BMP9^32d^) was briefly treated with trypsin (2 min) to isolate cells for expansion in monolayer culture. This dissociation procedure removed the majority of the fibrous layer without dissociating the underlying cartilage (Fig. S4A,B). The isolated cells (P3*^D8^*/F1) in monolayer culture had a cell morphology similar to that of P3*^D8^* clonal cells (Fig. S4C,D) and displayed a similar passage time in culture. Following differentiation using the BMP2-SA^4d^→BMP9^14d^ protocol, P3*^D8^*/F1 cells displayed a chondrogenic response identical to that of P3*^D8^* cells (Fig. S4E). AC maturation of P3*^D8^*/F1 cells was analyzed in extended cultures (BMP2-SA^4d^→BMP9^40d^) and histological analysis (*n*=4) identified central chondrocytes with enhanced collagen production relative to that of peripheral chondrocytes, and the differentiation of an encapsulating fibrous layer (Fig. 5F). Immunohistochemical analysis (*n*=3) identified chondrocytes expressing Cilp and Acan (Fig. 5G,H). Prg4-positive chondrocytes were observed scattered within the regenerated cartilage and also in the fibrous layer (Fig. 5I). ColI-expressing cells were restricted to the fibrous tissue layer (Fig. 5J). These studies indicate that the P3*^D8^*/F1 cells of the fibrous layer contain articular chondroprogenitor cells with characteristics identical to those of P3*^D8^* clonal cells. To confirm this conclusion, fibrous layer cells from P3*^D8^*/F1-regenerated AC were isolated (P3*^D8^*/F2), expanded and tested for chondrogenic potential. Using the BMP2-SA^4d^→BMP9^14d^ protocol (*n*=3), P3*^D8^*/F2 cells were found to display a chondrogenic response similar to that of P3*^D8^* and P3*^D8^*/F1 cells (Fig. S4F). These results demonstrate that articular chondroprogenitor cells are sequestered into an encapsulating fibrous tissue layer during BMP2-SA^4d^→BMP9^40d^-induced regeneration of AC.

### *In vivo* maintenance and stability of *in vitro*-differentiated AC

**Fig. 6.**
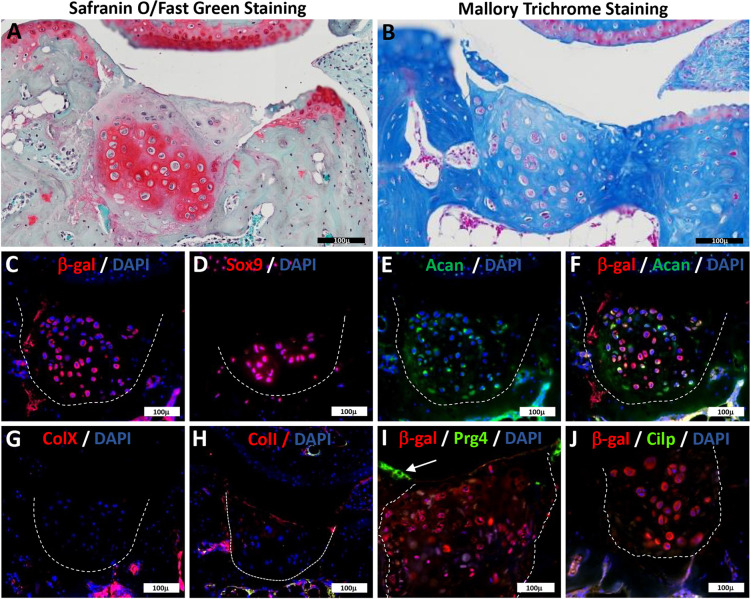
**Implantation of P3*^D8^*-derived articular cartilage into an acute joint defect.** P3*^D8^* cells differentiated into AC using the BMP2-SA^4d^→BMP9^40d^ protocol were implanted into a surgically created MtP joint defect and analyzed after 90 days (*n*=12). (A,B) Histological assessment included staining with Safranin O/Fast Green to identify production of glycosaminoglycan (A) and Mallory's trichrome to assess cartilage phenotype and production of collagens (B). (C-H) Adjacent serial sections of a single sample. (C,E,F) Double immunostaining for β-gal (C) and Acan (E) identified cells engrafted in the tissue co-expressing the lineage marker and the articular protein Acan (F). The images in C,E,F represent the same section. (C) Immunohistochemical staining for β-gal identified cells in the engrafted tissue. (D) Most engrafted cells expressed Sox9. (E) Engrafted cells retained expression of Acan. (F) Double immunostaining for β-gal (C) and Acan (E) identified engrafted cells co-expressing the lineage marker and the articular protein Acan. (G) Immunostaining for the hypertrophic chondrocyte marker ColX was negative. (H) Immunostaining for the fibrocartilage marker ColI was negative. (I) Double immunostaining for β-gal and Prg4 indicated that engrafted (β-gal^+^) cells did not retain expression of Prg4, whereas Prg4 expression (arrow) was prominent in the superficial layer of the host articular cartilage. (J) Double immunostaining for β-gal and Cilp indicated that engrafted (β-gal^+^) cells did not retain expression for Cilp. The dotted white lines in C-J indicate the interfaces of grafted cells and host tissues. All antibody studies: *n*=3. All scale bars: 100 µm.

To determine the *in vivo* stability of P3*^D8^*-regenerated AC using the BMP2-SA^4d^→BMP9^40d^ protocol, we transplanted cartilage into an acute metatarsal-phalangeal (MtP) joint defect. The central region of AC plus subchondral bone was removed from the phalangeal side of the joint and *in vitro*-regenerated AC tissue was immediately implanted into the defect as described (Yu et al., 2022). Immunodeficient SCID-NOD mice were used as hosts to minimize graft rejection. The P3*^D8^* clonal line was derived from the *lacZ*^+^ P3 cell line (Wu et al., 2013), allowing for the identification of engrafted cells by immunohistochemical staining for β-galactosidase (β-gal). The cartilage tissue was structurally firm and resilient to pressure when cutting and handling in preparation for transplantation. In preliminary studies, cartilage tissues generated using a BMP2-SA^4d^→BMP9^14d^ or BMP2-SA^4d^→BMP9^26d^ protocol failed to maintain AC characteristics following engraftment. After a 90-day implantation period, host joints were collected and processed for histological and immunohistochemical analyses. Previously we determined that control non-chondrogenic implants failed to survive transplantation, whereas all implants of P3-derived hyaline cartilage retained hyaline characteristics but did not develop AC characteristics (Yu et al., 2022). Thus, it was important to determine whether implants maintained AC characteristics. A total of 12 experimental joints containing BMP2-SA^4d^→BMP9^40d^-differentiated AC from P3*^D8^* clonal cells were analyzed and all implants survived the 90-day implantation period. Safranin O/Fast Green staining showed that the joint defect was filled with cartilaginous tissue rich in glycosaminoglycans in all 12 samples (Fig. 6A). The boundary between implanted cartilage and surrounding host tissues was apparent in Safranin O/Fast Green-stained sections, but less apparent in Mallory's trichrome-stained sections (Fig. 6B). Immunostaining (*n*=3) for β-gal demonstrated survival of implanted P3*^D8^* cells (Fig. 6C) and verified the graft/host boundary that was apparent in histological sections. Almost all engrafted chondrocytes expressed Sox9 (Fig. 6D), indicating an enhanced level of expression compared with that in the engrafted tissue and similar to that of maturing AC (Fig. 4H; Fig. S1C). Double immunostaining for β-gal and Acan (Fig. 6C,E,F) demonstrated that implanted P3*^D8^* cells retained Acan expression established in culture. ColX was not expressed (Fig. 6G) and there were a few cells in the periphery of the graft region that were positive for ColI (Fig. 6H). The β-gal-positive engrafted cells did not retain expression of Prg4 (Fig. 6I) or Cilp (Fig. 6J), thus not all articular chondrocyte marker proteins are retained after engraftment. Although Prg4 is normally expressed on the digit joint surface (Fig. 6I, arrow), whereas Cilp is not. These results indicate that following surgical transplantation, AC differentiated from P3*^D8^* clonal cells survived, retained some articular chondrocyte characteristics (Sox9 and Acan expression), and did not differentiate into hypertrophic cartilage (ColX expression) or fibrocartilage (ColI expression).

## DISCUSSION

Damaged AC does not undergo a regenerative response *in vivo* but triggers a pathological response that leads to progressive tissue loss and impaired joint function. Although AC does not normally regenerate, AC regeneration occurs in conjunction with induced joint regeneration *in vivo* following sequential treatment of digit amputation wounds with BMP2 and BMP9 (Yu et al., 2019), and hyaline cartilage regeneration *in vitro* is induced using an organoid culture model of BMP9-treated digit fibroblasts (Yu et al., 2022). The chondrogenic response to BMP9 was used to identify clonal lines of digit fibroblasts that displayed chondrocyte-specific progenitor properties, and an articular chondrocyte-specific progenitor line was used to establish an organoid culture protocol to differentiate into AC. AC was identified based on numerous criteria, including: (1) external and histological appearance, (2) a layered organization of articular chondrocytes, (3) expression of AC zone-specific matrix proteins, (4) a comparative genome-wide transcriptome analysis with mature AC and (5) the absence of hypertrophic chondrocytes. Moreover, histological and some immunohistochemical characteristics of *in vitro*-regenerated AC were maintained after transplantation *in vivo*. A crucial component of this study is the discovery that induced differentiation of AC is coupled with the differentiation of an encapsulating fibrous connective tissue layer that contains articular chondrocyte-specific progenitor cells. The sequestration of progenitor cells during differentiation foreshadows future injury responses – an attribute selected for during evolution and indicative of an endogenous regenerative program. Other musculoskeletal tissues that display strong regenerative capabilities include muscle and bone, and, in both cases, the regenerative response involves cell differentiation coupled with the sequestration of progenitor cells available for future injury. During muscle regeneration, muscle stem cells (satellite cells) are sequestered and available for repeated rounds of muscle repair (reviewed by Baghdadi and Tajbakhsh, 2018). Amputation of the rodent digit tip represents a model for epimorphic bone regeneration involving multiple progenitor populations (reviewed by Storer and Miller, 2020) and regeneration following re-amputation provides evidence of progenitor sequestration during bone regeneration (Dolan et al., 2019, 2022). What makes induced AC regeneration unique is the fact that it is a non-regenerative tissue with a dormant regenerative program that remains silent following acute joint injury. We have demonstrated that this dormant program can be activated in progenitor cells (see below) and, following expansion, BMP2-enhanced SA and BMP9-induced chondrogenesis, culminates in the regeneration of AC. The characterization of this dormant regenerative program represents a novel interface between regeneration biology and regenerative medicine (Dolan et al., 2018).

One remarkable finding from these studies is that the regeneration protocol induces both the differentiation of articular chondrocytes and a non-chondrogenic connective tissue layer that encapsulates the AC. The connective tissue layer is well defined histologically and appears similar to the stromal layer that is associated with AC *in vivo* and known to contain chondroprogenitor cells (Tong et al., 2019). Likewise, enzymatic digestion of the encapsulating layer results in the release of cells identified as articular chondrocyte-specific progenitor cells based on induced regeneration of AC. The connective tissue layer is heterogeneous, with cells expressing ColI intermingled with cells immunopositive for Prg4. *Prg4* expression identifies articular chondrocyte progenitor cells during development (Decker et al., 2017; Kozhemyakina et al., 2015; Li et al., 2017) and is required for BMP9-stimulated joint regeneration *in vivo* (Yu et al., 2019), thus the presence of Prg4 expressing cells may be linked to progenitor cell availability in the regenerated connective tissue layer. The cells of this connective tissue layer appear to be quiescent and unresponsive to BMP9 as they are readily exposed to BMP9 present in the differentiation medium. After brief enzymatic dissociation, however, isolated cells (P3*^D8^*/F1) display P3*^D8^* progenitor characteristics: aggregation enhanced by BMP2, articular chondrocyte differentiation induced by BMP9, and the sequestration of progenitor cells within an encapsulating connective tissue layer. The transition of connective tissue fibroblast from quiescence to proliferation occurs following enzymatic dissociation and appears to represent an activation response that initiates the regeneration process. A similar cell activation associated with histolytic degradation of tissues following digit amputation (Dawson et al., 2016; Fernando et al., 2011) can be inferred based on the observation that BMP9-induced chondrogenesis requires amputation injury (Yu et al., 2019, 2022). Thus, the data support a model in which the enzymatic dissociation of cells *in vivo* or *in vitro* serves to activate progenitor cells by mediating their release from differentiated tissues to enable participation in a regenerative response.

Cell-based therapies to engineer AC have been investigated for almost 30 years, but their clinical effectiveness remains limited (Correa and Lietman, 2017; Medvedeva et al., 2018). Significant challenges include: (1) the identification of a cell source, (2) an efficient methodology for articular chondrocyte differentiation and (3) maintaining AC characteristics following transplantation *in vivo* (Brittberg et al., 1994; Correa and Lietman, 2017; Demoor et al., 2014; Medvedeva et al., 2018). These challenges are not trivial as the choice of cell source is linked to the differentiation protocol, and multiple cell sources for engineering AC have been identified. For example, differentiating AC from induced pluripotent stem cells or embryonic stem cells requires a protocol that mimics developmental chondrogenesis (Craft et al., 2015; reviewed by Nakayama et al., 2020), whereas differentiating cartilage from mesenchymal stem cells (Johnstone et al., 1998; Mackay et al., 1998) or skeletal stem cells (Chan et al., 2015) requires a direct differentiation protocol. Using a combination of these strategies, we have established an organoid culture model to mimic early stages of embryonic chondrogenesis (condensation), followed by BMP9 induction (Yu et al., 2022) to differentiate articular chondrocytes from an adult progenitor cell. First, fibroblasts represent an important cell type for successful regeneration of cartilage *in vivo*. Fibroblasts play a key role in controlling amphibian limb regeneration and can trans-differentiate into chondrocytes *in vivo* and *in vitro* in both amphibians and mammals (Kragl et al., 2009; Muneoka et al., 1986; Nacu et al., 2013; Yu et al., 2022). In mammals, culture conditions that promote fibroblast expansion while retaining regenerative competence and chondrogenic potency have been established (Jiang et al., 2002; Wu et al., 2013; Yu et al., 2022), and clonal chondroprogenitor lines can be derived and expanded using these culture conditions. Second, when digit-derived P3 fibroblasts are cultured in suspension, they self-aggregate and differentiate into hyaline cartilage when induced with BMP9 (Yu et al., 2022). The idea that SA of cultured cells mimics chondrogenic condensation is supported by the finding that BMP2 enhances SA as it does chondrogenic condensation during development (Giffin et al., 2019). This SA step is uniquely different from traditional engineering approaches (e.g. forced aggregation by centrifugation) as cells can self-organize prior to induced differentiation. The final phase of AC regeneration is BMP9-induced differentiation of cell aggregates. BMP9 is a potent inducer of chondrogenesis, and enhanced expression of chondrogenic genes occurs within 24 h of treatment (Morgan et al., 2020; Yu et al., 2019, 2022). The chondrogenic response induced by BMP9 is, however, dependent on the progenitor clone; the P3*^D8^* clone differentiates into AC, whereas the P3*^E3^* clone differentiates into hypertrophic cartilage. Differential expression analysis of cartilage induced by our protocol identified hypertrophic chondrocyte-specific genes upregulated in P3*^E3^* clonal cells (*Ihh*, *Mmp13* and *Ror2*) (Rim et al., 2020; St-Jacques et al., 1999; Thorup et al., 2020) and articular chondrocyte-specific genes upregulated in P3*^D8^* clonal cells (*Cilp*, *Enpp2*, *Osr1* and *Sfrp2*) (Bernardo et al., 2011; Gao et al., 2011; Leimeister et al., 1998; Lorenzo et al., 1998; Ozpolat et al., 2012). Overall, this organoid approach establishes a previously unreported protocol for induced cartilage regeneration and identifies chondrocyte-specific progenitor cell types as a crucial variable for determining the type of cartilage regenerated.

AC consists of chondrocytes arranged into layers that range from the small chondrocytes of the superficial zone to progressively larger chondrocytes of the middle and deep zones and to the hypertrophic chondrocytes of the calcified zone (Carballo et al., 2017). Cell lineage studies demonstrate that all layers of articular chondrocytes are derived developmentally from cells of the superficial zone (Decker et al., 2017; Kozhemyakina et al., 2015). There is evidence that layer-specific morphogenesis occurs by clonal expansion (Li et al., 2017) as well as by differential cell growth and rearrangements that are niche specific (Decker et al., 2017). The regenerative potential of P3*^D8^* clonal cells is restricted to articular chondrocytes of the superficial, middle and deep zones but not the calcified zone, suggesting a stable chondroprogenitor responsible for forming the upper zones during AC regeneration. It is intriguing that regenerated articular chondrocytes are organized into concentric layers with small chondrocytes that are similar to middle-zone chondrocytes and located central to large chondrocytes that are similar to deep-zone chondrocytes. However, a cell layer similar to the superficial zone does not form and cells expressing Prg4 are randomly scattered throughout the regenerated cartilage. As P3*^D8^* cells are clonally derived, the organization of articular chondrocytes into distinct layers during regeneration likely involves extrinsic factors that may provide insight into how articular chondrocytes are organized *in vivo*. One possibility is that the chondrocyte phenotype is influenced by a differential response to nutrient and/or oxygen availability within the avascular layers of AC that parallels a response observed during chondrogenesis *in vitro* (Anderson et al., 2018). Such physiological gradients could influence gene expression as well as chondrocyte enlargement, resulting in distinct size differences across the AC zones. Another possibility is that physical interactions that drive morphogenesis during embryogenesis, such as differential cell/matrix adhesion, may play a role in organizing articular chondrocytes into distinct zones during development and regeneration (Foty and Steinberg, 2013; Walma and Yamada, 2020).

AC progenitor cells are present in mature AC (Johnstone et al., 2020) but do not participate in *in situ* repair following acute injury. The availability of endogenous progenitor cells is indicative of an unrealized regenerative potential and the *in vitro* evidence indicates that a dormant regenerative response can be stimulated extrinsically by modification of the culture conditions. Reports of induced regeneration (1) by sequential treatment with BMP2 and BMP9 at digit amputations (Yu et al., 2019), (2) by co-treatment with BMP2 and a VEGF antagonist following acute AC injury (Murphy et al., 2020), or (3) by factors present in synovial fluid (Chau et al., 2022; Miura et al., 2020) provide evidence that AC regeneration *in situ* is possible. Together, these *in vitro* and *in vivo* studies represent encouraging support for the eventuality of stimulating AC regeneration *in situ* and point to the identification of endogenous articular chondrocyte progenitor cell types coupled with specific pro-regenerative modification of the healing environment as key steps toward clinical application.

## MATERIALS AND METHODS

### Animal studies

The NOD.CB17-*Prkdc^scid^*/J (SCID-NOD) mouse strain (*Mus musculus*), purchased from The Jackson Laboratory and bred in house at the Texas Institute of Genomic Medicine, was used as the host for tissue transplantation studies. The metatarsal-phalangeal (MtP) joint defect was surgically created in adult SCID-NOD mice as previously described (Yu et al., 2022). The MtP joint was contracted ventrally, and a longitudinal skin incision was made to expose the joint capsule. The proximal joint surface of the first phalangeal element (P1) was accessed via a dorsal incision of the joint capsule and an acute defect was created in the P1 joint surface at the central distal groove. The acute defect extended through the AC layer and subchondral bone into the P1 bone marrow. For tissue implantation, samples were prepared in advance to approximate the size of the MtP defect and maintained on ice. Histological analysis of unused samples validated the implanted cartilage phenotype. Implanted chondrogenic samples are hard and can be compressed to fit snugly into the acute wound site. The surface of the implant was aligned with the joint surface and straightening the digit maintained the position of the implant during healing. The joint capsule and the overlying skin were closed with 10-0 sutures (Ethicon). All animal experiments and techniques used were compliant with the standard operating procedures and approved by the Institutional Animal Care and Use Committees at the College of Veterinary Medicine and Biomedical Sciences at Texas A&M University.

### Clonal cell isolation and culture

β-galactosidase (*lacZ*)-expressing P3 (*lacZ^+^* P3) fibroblasts were cultured and expanded on fibronectin-coated dishes in mesenchymal stromal cell medium (Jiang et al., 2002) containing 2% FBS (Gibco) (basal medium) and supplemented with EGF (R&D Systems), PDGF (R&D Systems) and LIF (Millipore) (expansion medium) as described previously (Wu et al., 2013). Passage 9 *lacZ*^+^ P3 fibroblasts were used to generate clonal cell lines, and monolayer cultures were used to collect conditioned medium to aid in the cloning process. To generate single-cell-derived clones, P3 fibroblasts were serial diluted to a concentration of 5 cells/ml and 200 µl aliquots were plated into each well of a 96-well plate (08-772-2C, Thermo Fisher Scientific). Cells were cultured using a 1:1 mixture of expansion medium and conditioned medium. The wells that contained a single cell were identified visually, expanded and stored in liquid Nitrogen for future use. The single-cell-derived clones on 96-well plates were designated as clonal passage (C-passage) 0, and all experiments were carried out with cells less than C-passage 6. Studies to re-derive progenitor cells were carried out by enzymatic dissociation (0.25% trypsin, 2 min) of differentiated cartilage. Dissociated cells were filtered with a 100 µm cell strainer (Corning, 431752) and plated onto fibronectin-coated dishes in expansion medium. At 70-80% confluence, cells were either dissociated and frozen in liquid nitrogen or expanded for chondrogenesis studies. The remaining undigested cartilage tissue was fixed, sectioned and stained to determine the effects of the brief trypsin treatment.

### Differentiation assay

For chondrogenesis studies, all cells were grown in expansion medium and collected at 70-80% confluency. To assay differentiation of pellet cultures, cells (2.5×10^5^) in basal medium (0.5 ml) were centrifuged (150 ***g***, 5 min) in 15 ml polypropylene tubes and cultured in basal medium supplemented with BMP9 (50 ng/ml, R&D Systems) (BMP9 differentiation medium) for 21 days. The medium was changed twice a week until the end of culture period. To assay differentiation of self-aggregated cultures, 2.5 ml of cells (1.0×10^5^ cells/ml) were resuspended in basal medium in a Petri dish (45 mm, Thermo Fisher Scientific) to minimize substrate attachment for 4 days and to encourage cell aggregation as described previously (Yu et al., 2022). To enhance the aggregation response, BMP2 (50 ng/ml, R&D Systems) was added to the basal medium (BMP2 aggregation medium) for the 4-day period. After aggregation, the medium was changed to BMP9 differentiation medium (changed twice weekly) until the end of the culture period.

### Histology and immunochemistry

*In vitro*-differentiated tissues were fixed with Z-fix (Anatech, 6269) and *in vivo*-implanted tissues were fixed with Z-fix and decalcified using Decalcifier I (Surgipath, Leica 3800400). Samples were then processed for paraffin histology and immunohistochemistry as previously described (Yu et al., 2022). For histological analysis, the samples were stained with Mallory trichrome (Yu et al., 2022) or Safranin O/Fast Green (Schmitz et al., 2010). Immunohistochemical staining for β-gal, Sox9, ColI, Acan, Prg4 and Cilp was carried out using heat retrieval [citrate buffer (pH 6), 65°C, 20 h], and antigen retrieval for ColX immunostaining used 1% hyaluronidase in PBS (Sigma-Aldrich, H3506, room temperature, 30 min). Slides were treated in Protein Block Solution (Dako, X0909; room temperature, 1 h). Primary antibodies included anti-β-gal (chicken polyclonal antibody, Abcam, ab9361; 1:500), anti-Sox9 (rabbit polyclonal antibody, Abcam, ab26414; 1:500), anti-ColX (rabbit polyclonal antibody, Abcam, 58632; 1:500), anti-ColI (rabbit polyclonal antibody, Origene, R1038; 1:200), anti-Acan (rabbit polyclonal antibody, Millipore, AB1030; 1:300), anti-Prg4 (rabbit polyclonal antibody, LSBio, LS-B8236; 1:200) and anti-Cilp (rabbit polyclonal antibody, Novus Biologicals, NBP1-81667; 1:100). Secondary antibodies included Alexa Fluor 568 goat anti-rabbit IgG (Invitrogen; A11011, 1:500), Alexa Fluor 568 goat anti-chicken IgG (Invitrogen, A11041, 1:500) and Alexa Fluor 488 goat anti-rabbit IgG (Invitrogen, A11008, 1:500). Slides were counterstained with DAPI to label nuclei. Slides were imaged with an Olympus BX61 fluorescence deconvolution microscope utilizing SlideBook software (Intelligent Imaging Innovations, Denver, CO, USA). Details of immunostaining procedures have been described previously (Dawson et al., 2019; Han et al., 2008; Yu et al., 2012). Quantitation of immunostained sections was carried out as previously described (Brunauer et al., 2021).

### RNAseq

Total RNA was extracted from homogenized samples using the RNeasy Plus Micro Kit (Qiagen) following the manufacturer's recommended protocol. RNA samples were processed by BGI Genomics and analyzed for concentration, RNA integrity number and the 28S/18S ratio using an Agilent 2100 Bioanalyzer prior to sequencing. The 100-bp-length paired-end reads from cultures were generated using the BGISEQ-500 sequencing system at BGI Genomics, whereas the Sequence Read Archive (SRA) shared datasets were downloaded from the NCBI website [primary ACs: SRR9317861, SRR9317862 and SRR9317863 (Bekki et al., 2020); primary GP cartilage: SRR5036053, SRR5036054, SRR5036055 and SRR5036056, (Sabik et al., 2017)]. All reads were aligned and referenced against the *M. musculus* genome (UCSC version mm10) using ‘HTSAT2’ (Kim et al., 2015) and counted using ‘htseq-count’ (Putri et al., 2022). The normalization and DE analysis were processed by DESeq2 with the normalization method of ‘median of ratios’ running under a Galaxy instance (Love et al., 2014). All datasets were normalized together and subsequently used for the respective DE analysis. The significant DE genes were defined by a cutoff of log_2_FC≥|1.5| and FDR *P*-value<0.05. The volcano plot analysis, correlation analysis and heatmap coupled with clustering analysis were performed and visualized by R/R studio with the packages ‘gplots’ (https://github.com/talgalili/gplots), ‘ggplot2’ (https://ggplot2.tidyverse.org), ‘corrplot’ (https://github.com/taiyun/corrplot), ‘heatmap.plus’ (https://github.com/cran/heatmap.plus) and ‘RColorBrewer’ (https://cran.r-project.org/web/packages/RColorBrewer/index.html). Correlation analysis was performed on the respective sets of DE using the averages (across each one of the four phenotypes) of the normalized gene expression values.

## Supplementary Material

10.1242/develop.201894_sup1 Supplementary information

Table S1. The list of 5,142 differentially expressed genes (log2(FC) ≥ | 1.5 | and FDR P-value < 0.05) resulting from a comparison of RNAseq datasets for articular cartilage and growth plate cartilage.
