# Supplementary material for: Induced regeneration of articular cartilage – identification of a dormant regeneration program for a non-regenerative tissue
Source: Development. 2023 Nov 8;150(21):dev201894. doi: 10.1242/dev.201894 (PMC10651102; doi:10.1242/dev.201894)
Supplement: Supplementary information [file develop-150-201894-s1.pdf]

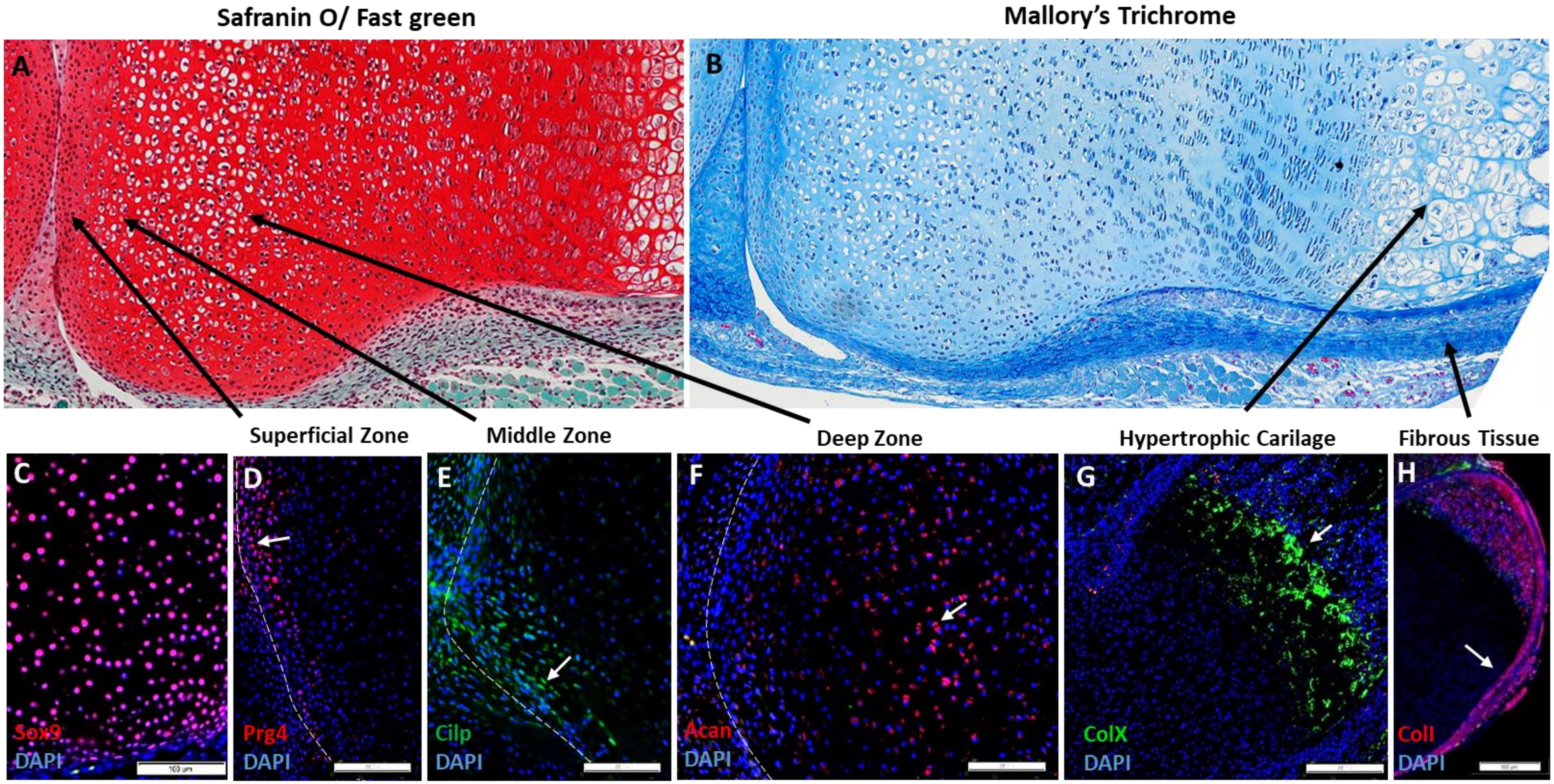

Fig. S1. Histological and immunohistochemical staining of the postnatal day 7 neonatal knee. A) Longitudinal section of the joint region stained with Safranin O/fast green identifies chondrocytes with surrounding extracellular matrix rich in glycosaminoglycans (red). B) Longitudinal section of the joint region stained with Mallory's trichrome shows a gradient of chondrocyte size in the joint forming region and hypertrophic chondrocytes of the growth plate. C) Cells of the joint forming region display uniform expression of Sox9. D) Chondrocytes of the superficial zone (arrow) are small and express Prg4. E) Middle zone chondrocytes (arrow) are small with lacunae and express Cilp. F) Chondrocytes of the deep zones (arrow) have large lacunae and express Acan. The dashed line in D-F outlines the articular boundary in the joint. G) Columns of hypertrophic chondrocytes of the growth plate (arrow) express ColX. H) Surrounding fibrous connective tissue (arrow) are non-chondrogenic and are positive for ColI expression. Scale bars: C = 100 µm; D-G = 200 µm; H = 300 µm.

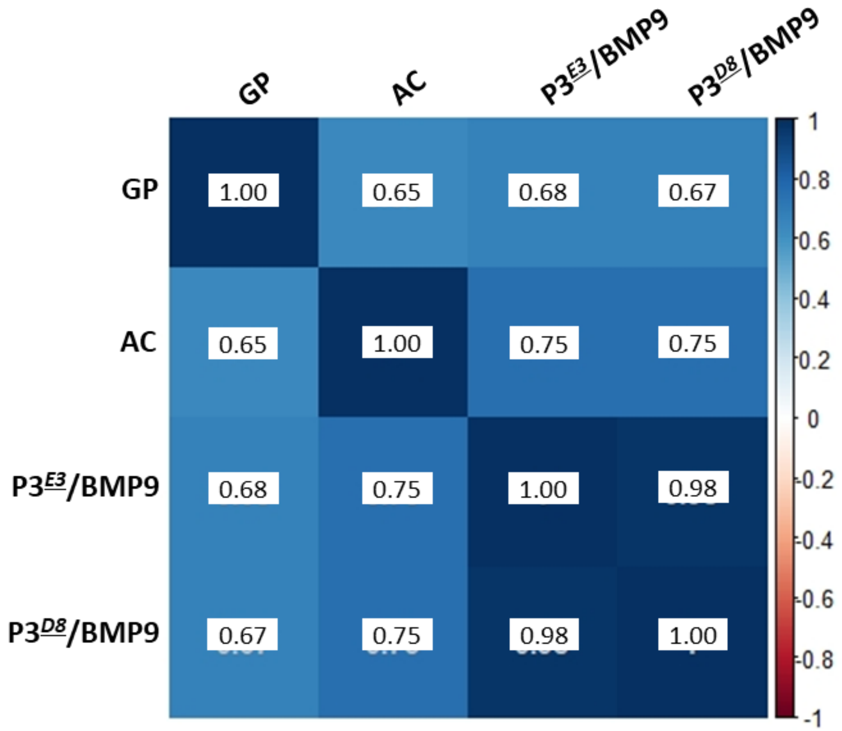

Fig. S2. Complementary correlation analysis using all of the DE genes resulting from the comparison of articular cartilage (AC) and growth plate (GP) cartilage samples detected a positive correlation between AC and GP.

Development • Supplementary information

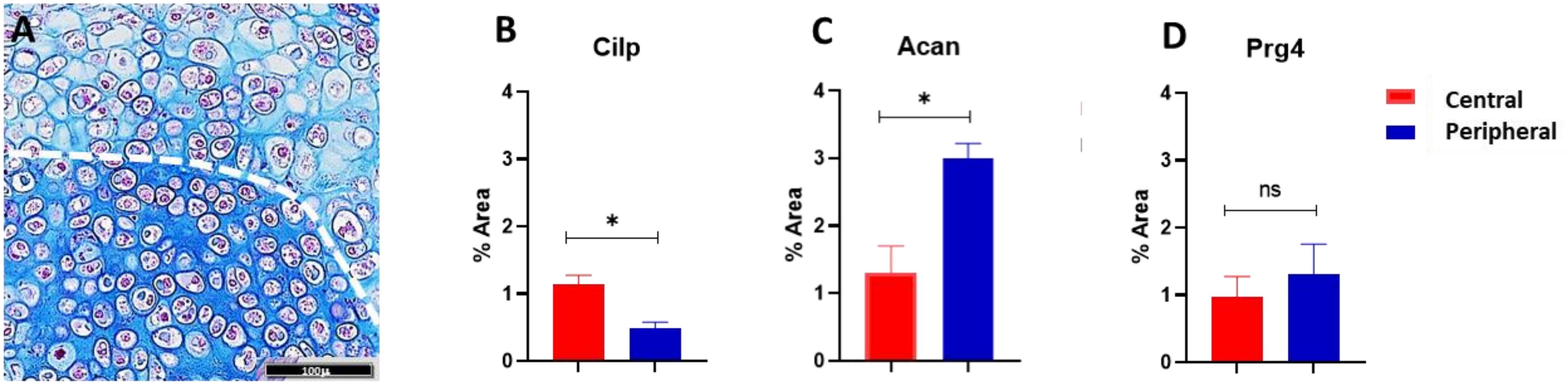

Fig. S3. Layered organization of chondrocytes in regenerated articular cartilage. A) Mallory's trichome stained sections were used to delineate central cartilage from peripheral cartilage based on chondrocyte size and collagen matrix production (n=6). **B)** Immunostaining of adjacent sections for expression of Cilp (n=3) was analyzed to determine if expression was regionally controlled. An unbiased imaging analysis of Cilp expression indicated that expression was statistically enhanced (mean ± SEM) in the central chondrocytes compared to the peripheral chondrocytes. **C)** Immunostaining of adjacent sections for expression of Acan (n=3) was analyzed to determine if expression was regionally controlled. An unbiased imaging analysis of Acan expression indicated that expression was statistically enhanced (mean ± SEM) in the peripheral chondrocytes compared to the central chondrocytes. **D)** As a control, Prg4 expressing cells were found to be evenly distributed (mean ± SEM) between central and peripheral cartilage regions.

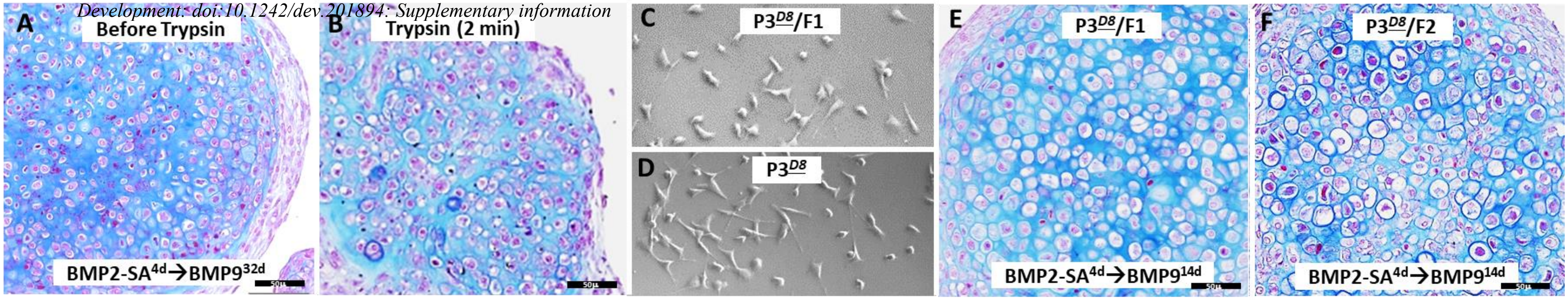

Fig. S4. Fibrous layer cells contain articular chondrocyte progenitor cells. A,B) P3$^{\underline{D8}}$ differentiated cartilage was treated with trypsin for 2 minutes to collect cells from the fibrous layer (n=3). Tissue prior to trypsin treatment (A) shows the fibrous tissue layer intact and after trypsin treatment (B) most of the cell layer is disrupted which cartilage tissue remains intact. C,D) The cell morphology of P3$^{\underline{D8}}$/F1 cells (C) is similar to P3$^{\underline{D8}}$ cells (D). E) P3$^{\underline{D8}}$/F1 cells were treated using the BMP2-SA^{4d}→BMP9^{14d} protocol to determine if cartilage regenerated (n=3). Mallory's trichrome staining of P3$^{\underline{D8}}$/F1 regenerated indicated a robust chondrogenic response. F) P3$^{\underline{D8}}$/F1 cells were used to form mature articular cartilage using the BMP2-SA^{4d}→BMP9^{40d} protocol and P3$^{\underline{D8}}$/F2 cells were isolated by brief trypsin treatment. After treatment using the BMP2-SA^{4d}→BMP9^{14d} protocol, P3$^{\underline{D8}}$/F2 cells displayed a robust chondrogenic response. Scale bar A,B,E,F = 50 µm.

Table S1. The list of 5,142 differentially expressed genes ($\log_2$(FC) ≥ │1.5│ and FDR P-value < 0.05) resulting from a comparison of RNAseq datasets for articular cartilage and growth plate cartilage.

Available for download at
https://journals.biologists.com/dev/article-lookup/doi/10.1242/dev.201894#supplementary-data

Development • Supplementary information

Table S2. Differentially Expressed Genes by Cartilage Derived from P3$^{\underline{D8}}$ and P3$^{\underline{E3}}$ cells

P3$^{\underline{D8}}$ > P3$^{\underline{E3}}$: *1700109K24Rik, 1810011O10Rik, 1810030O07Rik, 2310015D24Rik, 2610035F20Rik, 2700081O15Rik, 2810403A07Rik, 2810433D01Rik, 4732440D04Rik, 4930412C18Rik, 4930579G18Rik, 4933427D14Rik, 5430403N17Rik, 5730480H06Rik, 9330117O12Rik, A230028O05Rik, A230050P20Rik, A230083G16Rik, A730020E08Rik, A830019L24Rik, A930038B10Rik, Abr, Acat3, Acpp, Adamts12, Adamts2, Adamts5, Adamts6, Adamtsl2, Adssl1, AI480526, Akr1c14, Alox8, Amn1, Ang2, Apeh, Apol6, Aqp1, Aqp5, Arhgef2, Arhgef28, Arhgef40, Arid4b, Atp1a2, Atp1b1, Atp8b1, Avpr1a, B230354K17Rik, B4galt2, Baiap2, Baz1b, Bbs12, BC064078, Bcl6, Bcl6b, Bclaf1, Bdkrb1, Bend4, Bhlhe22, Bicc1, Birc2, Birc3, Blnk, Bmp1, Bmp2, Brpf3, C3, Cand2, Capg, Ccbe1, Ccdc157, Ccdc24, Ccl2, Ccl7, Ccl9, Ccnl1, Ccnl2, Ccser1, Cd28, Cdc37l1, Cdh24, Cdk13, Cdk14, Cercam, Cetn3, Cfap69, Chn2, Chrdl1, Chst10, Cilp, Cilp2, Cldn1, Clec14a, Clec16a, Clec2e, Clk1, Clk4, Clstn2, Cmya5, Cnrip1, Col3a1, Col6a3, Col8a1, Cpne3, Crabp2, Crlf3, Cry1, Cttn, Cxcl11, Cxcl12, Cxcr6, Cxxc4, Cyhr1, Cyth1, D030025P21Rik, D730005E14Rik, D830031N03Rik, D930048N14Rik, Dapk2, Dcn, Ddi2, Ddr2, Ddx17, Dennd2d, Desi2, Dexi, Dmrta1, Dnm3os, Doc2b, Dok3, Dpt, Dpyd, Dpysl2, Dpysl3, Dse, Dusp16, Dusp4, Dzip1l, E330033B04Rik, Egfr, Eif4e2, Eif4g1, Enpp2, Epb41l2, Epha2, Epn2, Ereg, F2r, Fam13b, Fam214a, Fam46b, Fam65a, Fam83h, Far1os, Farp1, Fastk, Fbln1, Fbxo22, Fdft1, Fendrr, Fgf10, Flt1, Fmr1, Foxf1, Foxred2, Foxs1, Gabre, Galnt12, Gapdhs, Gas1, Gbp5, Gdap10, Gjb4, Gm10710, Gm11783, Gm12338, Gm13056, Gm13157, Gm15411, Gm16576, Gm16976, Gm20544, Gm26705, Gm28523, Gm30177, Gm30510, Gm30956, Gm31012, Gm31066, Gm31077, Gm31078, Gm31512, Gm32219, Gm32404, Gm32817, Gm33153, Gm33473, Gm34389, Gm34589, Gm35824, Gm38469, Gm38592, Gm38675, Gm38850, Gm38914, Gm39668, Gm40121, Gm40468, Gm40725, Gm40755, Gm40841, Gm40960, Gm41032, Gm41159, Gm41280, Gm41420, Gm41428, Gm42127, Gm42259, Gm5454, Gm6556, Gm6566, Gm6634, Gnl2, Golga4, Gprin3, Gpsm1, Gspt2, Gstm2, Gtf2a2, Hacd3, Hhat, Hook3, Hoxd10, Hp1bp3, Hpgd, Hpse, Hs3st3a1, Hsf2, Hspb7, Htr2a, Ifnlr1, Il17rb, Inf2, Inmt, Itga11, Itga2, Itgb8, Itgbl1, Jak2, Jun, Kansl1l, Kansl3, Kat6b, Kcnj2, Kctd9, Kdm2a, Khdrbs1, Klhdc8a, Lancl3, Lima1, Lims1, Lman1l, Lmbr1l, Lmod1, LOC102633880, LOC102639958, LOC108167377, LOC108167518, LOC108167700, LOC108167917, LOC108167946, LOC108168297, LOC108168933, LOC108168980, Lrrcc1, Lyve1, Mafb, Malrd1, Mamdc2, Mapk1ip1, Mark2, Masp1, Mbd4, Mblac2, Mbtd1, Mcm6, Megf10, Megf6, Mepe, Metap1d, Mettl13, Mfhas1, Mical2, Mir7652, Mmp9, Mn1, Mnt, Mprip, Msx2, Mt2, Mtss1l, Mturn, Murc, Mx1, Mxra7, Myo10, Myo9b, Myoc, Myzap, Naalad2, Ncapd3, Ncbp1, Net1, Nkd2, Nktr, Nrf1, Nrn1, Nuak1, Numbl, Olfml2a, Omd, Osr1, Palm2, Pde1b, Pde3a, Pdgfra, Penk, Pgam2, Pgm5, Phf11d, Phldb1, Pik3r1, Platr22, Plek, Pnisr, Pogk, Ppm1l, Prkg1, Proz, Prpf38b, Prrx1, Psg20, Pstpip2, Ptchd1, Ptgfr, Ptn, Pygm, Rad54l2, Rasa1, Rasl11a, Rbm3, Rbm39, Rbp1, Rbpj, Rc3h1, Reep1, Rel, Rem1, Rgs4, Rgs5, Rnf113a2, Rnf145, Rnf39, Rxfp3, S100a8, Sash1, Scx, Sema3a, Sema7a, Serf2, Setbp1, Sfrp2, Sh3bp4, Sh3rf3, Shb, Slc1a3, Slc25a27, Slc25a36, Slc25a37, Slc38a9, Slc3a2, Slco1a1, Slfn2, Smad3, Smchd1, Smpd4, Sntb1, Snx27, Snx30, Sod3, Sox21, Spire2, Spp1, Spred2, Sptbn1, Srpk1, Srrt, Srsf11, Srsf4, Stat6, Stk11, Stk38, Stmn2, Sugct, Svep1, Syncrip, Syngap1, Syt13, Taf8, Taz, Tbce, Tcte2, Tgfbr3, Thbs4, Timm21, Tm4sf1, Tmem156, Tmem200b, Tmem35, Tnfrsf1b, Tnfsf10, Tpm2, Tram1l1, Trim11, Trim2, Trp63, Trps1, Tspan13, Ttc28, Ttc9, Tubgcp5, Ubap2l, Ugt1a6a, Ugt1a6b, Ugt1a7c, Uhrf1bp1l, Unkl, Usp18, Usp40, Vegfc, Vezf1, Whrn, Wipf1, Wnt10a, Wsb1, Xcr1, Xdh, Zadh2, Zbed6, Zbtb5, Zcchc24, Zfp157, Zfp266, Zfp275, Zfp367, Zfp36l1, Zfp449, Zfp948, Zic2, Zkscan3, Zranb2*

P3$^{\underline{E3}}$ > P3$^{\underline{D8}}$: *1700008J07Rik, 1700047M11Rik, 1810024B03Rik, 2810468N07Rik, 3110079O15Rik, 4932435O22Rik, 9230110C19Rik, A2m, Adamts14, Adgrd1, Adm2, Adra1d, Adra2a, AI661453, AI662270, Aif1l, Akr1b7, Aldh1a3, Alx3, Amz1, Angpt4, Ankfn1, Ap1s3, Apcdd1, Aph1b, Apobr, Atf3, ATP8, AW112010, B230217C12Rik, Basp1, Batf3, BB557941, BC080695, Bc1, Bcam, Bcan, Bdnf, Bmp6, C2, C330021F23Rik, Ccdc8, Cd24a, Chac1, Chad, Cidea, Cisd3, Clec3a, Col11a1, Col14a1, Col9a2, COX3, Ctsc, Cybrd1, D630003M21Rik, Dlx4os, Dlx6os1, E330013P04Rik, Efnb2, Eif2s2, Eif3f, Eif4e3, Entpd2, Epha3, Epyc, Exoc3l2, Ezr, Fabp7, Fads6, Fau, Fbln7, Fjx1, Foxd1, Frzb, Galnt15, Gdf5, Gfap, Gimap6, Gjc3, Glp1r, Gm11478, Gm12070, Gm12174, Gm12273, Gm13552, Gm13848, Gm14434, Gm2225, Gm25380, Gm25432, Gm26633, Gm31213, Gm33844, Gm34865, Gm40019, Gm41213, Gm41556, Gm41844, Gm4705, Gm4786, Gm5148, Gm561, Gm5621, Gm5854, Gm5879, Gm6158, Gm6559, Gm6988, Gm7334, Gm8210, Gm8430, Gm8451, Gm8942, Gm9794, Got1, H2-DMb1, H2-DMb2, Hcn1, Hddc3, Hgf, Hist1h4h, Hist2h2bb, Hsd11b2, Icosl, Ihh, Irs1, Kcna5, Kcnmb4, Kcnq1ot1, Kctd4, Llph-ps2, LOC101055915, LOC101056032, LOC102631979, LOC102632292, LOC102634483, LOC105244208, LOC105247253, LOC108167548, LOC108167591, LOC108167751, LOC108167922, LOC108167926, LOC108168114, LOC108168154, Lpar4, Lrp2, Lrrc17, Lrrc4, Lypd3, Lyrm9, Lyz2, Mall, Mansc1, Map6d1, Mei4, Mertk, Metrn, Mia, Mir682, Mmp13, Mthfd2, Naip6, Napsa, ND3, Nfe2l3, Nhlrc1, Nog, Nos1ap, Npl, Nrp, Nrtn, Nup210, Nutf2-ps2, Palmd, Panx3, Paqr3, Pard6b, Pcdhga5, Pck2, Pi15, Pigq, Pigt, Pik3ap1, Plagl1, Podxl, Polr2l, Ppp1r15a, Ppp1r1b, Pycard, Pycr1, Rbms3, Rhox5, Rn7s1, Ror2, Rpf1, Rpl13a, Rpl13-ps6, Rpl14-ps1, Rpl17-ps8, Rpl27a, Rpl35, Rpl37, Rps11, Rps13, Rps15a, Rps15a-ps4, Rps15a-ps5, Rps15a-ps6, Rps16, Rps17, Rps26, Rps27rt, Rps3, Rps5, S100b, S1pr5, Sdhaf3, Sema3d, Sema3e, Shisa2, Slc13a4, Slc25a13, Slc25a33, Slc2a10, Slc35e3, Slc35g1, Smpdl3a, Snhg12, Snhg14, Snhg6, Snhg8, Sorl1, Sost, Sox8, Spon1, Srpx, Sstr2, Steap1, Stk32b, Stxbp2, Tcaf2, Tfrc, Tnfaip8l3, Trib3, Tspan18, Tst, Tubb3, Uchl1, Vax2, Wnt4, Zcchc5, Zfas1, Zfp941, Zfp971*
