# Supplementary material for: Induced regeneration of articular cartilage – identification of a dormant regeneration program for a non-regenerative tissue
Source: Development. 2023 Nov 8;150(21):dev201894. doi: 10.1242/dev.201894 (PMC10651102; doi:10.1242/dev.201894)
Supplement: Supplementary file 1 [file develop-150-201894_review_history.pdf]

Induced regeneration of articular cartilage - identification of a dormant regeneration program for a non-regenerative tissue

Yu-Lieh Lin, Ling Yu, Mingquan Yan, Katherine Zimmel, Osama Qureshi, Felisha Imholt, Tao Li, Ivan Ivanov, Regina Brunauer, Lindsay Dawson and Ken Muneoka
DOI: 10.1242/dev.201894

Editor: Kenneth Poss

Review timeline

Original submission:	18 April 2023
Editorial decision:	22 May 2023
First revision received:	1 September 2023
Accepted:	9 October 2023

Original submission

First decision letter

MS ID#: DEVELOP/2023/201894

MS TITLE: Induced regeneration of articular cartilage: Identification of a dormant regeneration program for a non-regenerative tissue

AUTHORS: Yu-Lieh Lin, Ling Yu, Mingquan Yan, Katherine Zimmel, Osama Qureshi, Felisha Imholt, Tao Li, Ivan Ivanov, Regina Brunauer, Lindsay A Dawson, and Ken Muneoka

I have now received all the referees' reports on the above manuscript, and have reached a decision. The referees' comments are appended below, or you can access them online: please go to BenchPress and click on the 'Manuscripts with Decisions' queue in the Author Area.

As you will see, the referees express considerable interest in your work, but have some critiques and requests for clarification, and they recommend a revision of your manuscript before we can consider publication. If you are able to revise the manuscript along the lines suggested, which may involve further experiments, I will be happy receive a revised version of the manuscript. Your revised paper will be re-reviewed by one or more of the original referees, and acceptance of your manuscript will depend on your addressing satisfactorily the reviewers' major concerns. Please also note that Development will normally permit only one round of major revision. If it would be helpful, you are welcome to contact us to discuss your revision in greater detail. Please send us a point-by-point response indicating your plans for addressing the referee’s comments, and we will look over this and provide further guidance.

Please attend to all of the reviewers' comments and ensure that you clearly highlight all changes made in the revised manuscript. Please avoid using 'Tracked changes' in Word files as these are lost in PDF conversion. I should be grateful if you would also provide a point-by-point response detailing how you have dealt with the points raised by the reviewers in the 'Response to Reviewers' box. If you do not agree with any of their criticisms or suggestions please explain clearly why this is so.

Reviewer 1

Advance summary and potential significance to field

© 2023. Published by The Company of Biologists under the terms of the Creative Commons Attribution License (https://creativecommons.org/licenses/by/4.0/).

The generation of articular cartilage for engraftment is a highly significant problem in regenerative biology. Identifying the optimal cell source for ex vivo articular cartilage generation is a key goal in the field. Previously, this group identified that P3 digit fibroblasts are a cell population that can be grown in the presence of BMP9 in organoid culture to resemble articular cartilage and survive engraftment into joint in vivo. The present study involves clonal selection of P3 fibroblasts and the refinement of culture protocols (BMP2->BMP9) using one of these clonal lines to further promote articular over hypertrophic cartilage fate in aggregate culture. RNA-seq was used for bulk gene expression characterization of two clonal P3 lines. Comparison of gene expression using only a small subset of genes from previous publicly available bulk-seq cartilage datasets was used to support phenotypic association of one clonal line with articular cartilage and the other with hypertrophic cartilage. While the title suggests the identification of a regeneration program, the results do not adequately support this claim. I suggest either additional characterization of the regenerative process in their model or a revised title to reflect the main findings of the manuscript.

Comments for the author

In Figure 1, with the clonal selection of P3 fibroblasts, how efficient is the differentiation protocol for articular cartilage vs the parent P3 line? The authors should quantify the percentage of Acan+;ColX- to Acan-;ColX+ clones to see whether clonal selection and/or BMP9 treatment results in any bias towards hypertrophic or articular fate.

In Figure 2, the morphology of the BMP2-treated P3E3 aggregates in J has an elongated almost fibroblast-like appearance instead of spherical morphology. What can account for this changed morphology? BMP2 has previously been reported to induce osteogenesis in culture; Is the BMP2 treatment inducing bone formation in P3E3 cells? Also, the P3E3 cells are described as having a "poor self-aggregation response" (Fig 2D, line 158). Is this due to decreased proliferation of these progenitors in response to BMP2 compared to P3D8 cells?

The images in Figure 2C and 2I are very close cropped. Could the authors show images representing the full cross section of these colonies and quantify and comment on how the overall size of these colonies and their differentiation profile compare to BMP2-SA4d->BMP940d? Staining and quantification of % cells expressing articular cartilage markers at the 14d phase (Prg4b, Cilp) should be used to show whether the additional maturation phase in culture is required for the induction of organization. Are the fibrous cells present in the colony periphery at 14d (it looks as if they are present surrounding the small cultures in Fig 2F)?

For the genome-wide analysis, it should be explicitly stated in the text and/or methods section which differentiation protocol and culture endpoint was used on the P3 lines prior to sequencing as they are marked only as P3/BMP9.

As the authors point out, previous studies have well established the similarities between the gene expression profiles of growth plate and articular cartilage particularly with overlapping zone-specific profiles (eg. Chao 2014). Given that the two datasets the authors use for their RNAseq references come from two different studies, it is concerning that the 30 genes used to define growth plate are primarily histone and RNA genes. Is this gene signature due to the biology of the tissue or due to batch effects or other technical difference in the data?
Validation in vivo or from the literature showing differential expression of these genes would assist in the interpretation of this comparison. Alternatively, would the correlation analysis using the entire gene set rather than the tope 60 DE genes or using previously published zonal laser-capture microdissection datasets from both the AC and GP (Chao 2014) provide a clearer picture of the molecular phenotype of these immature P3 derived chondrocytes.

In Figure 4C, the authors demonstrate differential gene expression in the two clonal populations. As these may be essential to driving the difference in cell phenotype or regenerative capacity, it would be helpful to either label on the volcano plot some of the key growth factor and chondrogenic genes discussed in the text and/or to include the fold change information for these key genes to illustrate how the expression compares between the two clonal lines. This would aid the interpretation of the significance of the discussed genes and provide more mechanistic insights into the differentiation phenotypes of the two clonal lines.

© 2023. Published by The Company of Biologists under the terms of the Creative Commons Attribution License (https://creativecommons.org/licenses/by/4.0/).

In addition to more details regarding the few chondrocyte genes listed, a more in-depth characterization of these genetic differences to show the global genetic changes in gene set enrichment (eg. TFs, growth factor signaling pathway activity) between the different differentiation trajectories could be used to more clearly define the regeneration program suggested by the title.

On line 275, the authors state that the P3D8-F1 isolated cells and P3D8 were similarly proliferative. Can the authors please provide these quantitative data.

The implant data in Figure 6 nicely shows persistent Safranin-O/Sox9+ graft survival after a 90-day period. The authors state on line 319 that these implants retain articular cartilage characteristics. To support defining the articular cartilage phenotype, it would be important to show whether the implants retain Prg4b and Cilp expression.

In general, the authors should include n numbers for all culture and staining experiments.

Reviewer 2

Advance summary and potential significance to field

Lin and colleagues have refined a cell culture organoid model using fibroblasts from the terminal phalanx (P3) of mice, to generate articular cartilage through sequential treatment with BMP2 and BMP9. This work appears to be a follow on study from their previous publication whereby the use of digit tip fibroblasts or their ability to generate articular cartilage has been previously described (Yu et al, 2022, Development). The authors however, have presented three new advances; (a) refinement of the culturing protocol which involves sequential treatment of fibroblasts with BMP2 and BMP9 leading to enhanced aggregation of ‘chondro-spheres’; (b) articular cartilage self-organises in culture generating a layer of encapsulating fibrous connective tissue; and (c) the transplantation of the newly generated articular cartilage was stable in vivo.
These findings represent an advancement to our current knowledge and would be of broad interest to developmental biologists and the regenerative medicine communities. The use of digit tip fibroblasts to generate cartilage is a novel and understudied area of research. Furthermore the study provides methodological advances in the generation of articular cartilage and characterisation of its potential for use in therapeutic applications. Since cartilage does not endogenously regenerate in mammals, its damage leads to impaired joint function and represents a significant burden on the health care system. Overall, I am supportive of the work described in this manuscript, however, would suggest some revisions as outlined below.

Comments for the author

- The authors use fibroblasts derived from the distal phalanx (P3) of the mouse digit tip. This could contain cells from the periosteum which contain a resident source of osteochondro-progenitors. Can the authors be certain they are not just amplifying clonal lines of these specific osteochondroprogenitors rather than fibroblasts having the potential to generate chondrocytes?

- Passage 9 P3 fibroblasts were used to generate the clonal cell lines. These cells have been cultured in MSC media for a prolonged period. Do you obtain similar results from cells taken directly from P3 digits?

- Can the authors comment on why their protocols to generate articular cartilage only correlates to in vivo articular cartilage with a p = 0.51? This is a relatively low correlation for similar cell types. A trajectory analyses using Monocle or RNA velocity might be more convincing here to show they are on their way to becoming articular cartilage (this would however require single cell RNA sequencing data rather than the bulk RNA sequencing data provided in the paper).

- Line 226 – The authors state that organized articular cartilage differentiates from P3D8 clonal cell cultures that has a structurally firm tissue that was resilient to pressure. How was this measured?

© 2023. Published by The Company of Biologists under the terms of the Creative Commons Attribution License (https://creativecommons.org/licenses/by/4.0/).

• Following transplantation of articular cartilage into the acute joint defect, there appears to be an abundance of Sox9 positive progenitor cells. This did not seem to be the case in vitro. Can the authors explain why Sox9 expression has increased?

• Was the transplanted articular cartilage functional? (i.e. Did the mice gain back normal gait for example?)

Additional Minor Comments
The authors use phrases (line 75) such as 'The chondrogenic response of P3 fibroblasts was explored by cloning cells and characterizing responsiveness to BMP9'. The use of the word cloning here is ambiguous (generally a molecular biology term). It would be better to state they generated clonal cell lines. This is also the case in the abstract.

Reviewer 3

Advance summary and potential significance to field

This manuscript describes a new organoid model to study the induction of cartilage tissue using BMP2+9. Several clonal mesenchymal cell lines could induce cartilage with different genetic signatures and alternative differentiation potentials. Induced cartilage could spontaneously form stratified layers of chondrocytes that could be implanted into surgical joint defects in vivo with retention of overall structure and immunoreactivity for several chondrocyte marker genes. This work represents an exciting step forward in understanding the potential for regeneration in mammalian tissues. The finding that chondroprogenitor cells are sequestered into the fibrous connective tissue layer that spontaneously forms in the BMP2+9 induction protocol is extremely interesting and reveals potential parallels with other regenerative contexts giving weight to the idea that mammals have more regenerative potential than previously thought. Overall, the manuscript is well written and represents a good resource for the field.

Comments for the author

Below are some comments that are designed to improve clarity in the manuscript.
1) In supplemental figure 1. The authors could improve clarity by indicating exactly what the dotted line represents in D-F.
Prg4+ chondrocytes butted up at the edge of the superficial zone.
Check the placement of the dotted line in E as Clip+ Chondrocytes also appear to be placed at boundary of superficial zone and middle zone.
2) The statement made on line 234 seems a little confusing when figure 4 is compared with supplemental figure 1. When one compares the marker gene localization with the developing postnatal neonatal knee cartilage the statement seems to be inconsistent.
The authors state that: "Overall, the BMP2-SA4d->BMP940d differentiated P3D8 cartilage was organized into concentric rings of chondrocytes displaying a gradient of increasing chondrocyte size and glycoaminoglycan production that parallels the middle and deep zones of maturing articular cartilage
(Supplemental Fig 1A). Immunostaining studies also supported this conclusion;"
However, in the p7 developing knee cartilage (Supp figure 1) they show:
C) Cells of the joint forming region display uniform expression of Sox9.
D) Chondrocytes of the superficial zone (arrow) are small and express Prg4.
E) Middle zone chondrocytes (arrow) are small with lacunae and express Cilp.
F) Chondrocytes of the deep zones (arrow) have large lacunae and express Acan.
G) Columns of hypertrophic chondrocytes of the growth plate (arrow) express ColX.
H) Surrounding fibrous connective tissue (arrow) are non-chondrogenic and are positive for ColI expression.
In organized articular cartilage differentiates from P3D8 clonal cell cultures they report:
-Sox9 expressing cells were not localized and abundant throughout the regenerated cartilage (Fig 4H)
-Prg4 expressing cells were found scattered throughout the cartilage and was not localized (Fig 4K; Supplemental Fig 3D).
-Cilp immune-positive cells were abundant in the central core of small chondrocytes while

© 2023. Published by The Company of Biologists under the terms of the Creative Commons Attribution License (https://creativecommons.org/licenses/by/4.0/).

-Acan immune-positive cells were most abundant in the peripheral region of larger chondrocytes (Fig 4F,G; 238 Supplemental Fig 3A-C).
-while ColX and ColI immune-positive cells are absent in the differentiated cartilage (Fig 4I,J).
It would be extremely helpful for the reader if the authors could write something to describe the contrast in these patterns. It is probably not unreasonable that the patterns would be different considering the extrinsic signals (or lack thereof) in the artificial culturing microenvironment, but these differences should be acknowledged and discussed.
Despite this pattern mismatch, it is interesting that regenerated articular chondrocytes are organized into concentric layers (possibly inverted?) and that further studies on extrinsic factors could provide important insights into this differentiation and organization.

Minor comments:
3) There may be a missing word on line 63. “Chondrocytes of the superficial zone are small, flattened 63 cells containing relatively low proteoglycan levels and are identified based on proteoglycan 4 (Prg4, also called lubricin)
expression of (Saito, 2022).”
4) In the discussion lines 395-398., they self-aggregate and differentiate hyaline cartilage when induced with BMP9 (Yu et al., 2022).
Is this supposed to read BMP2? (.BMP2 is supposed to induces the self-aggregation and BMP9 induces the differentiation, right?)

5) Supplementary table I seems to be missing from the manuscript PDF.

First revision

Author response to reviewers' comments

Detailed Response to Reviewer Comments
Reviewer 1 Advance summary and potential significance to field
The generation of articular cartilage for engraftment is a highly significant problem in regenerative biology. Identifying the optimal cell source for ex vivo articular cartilage generation is a key goal in the field. Previously, this group identified that P3 digit fibroblasts are a cell population that can be grown in the presence of BMP9 in organoid culture to resemble articular cartilage and survive engraftment into joint in vivo. The present study involves clonal selection of P3 fibroblasts and the refinement of culture protocols (BMP2->BMP9) using one of these clonal lines to further promote articular over hypertrophic cartilage fate in aggregate culture. RNA-seq was used for bulk gene expression characterization of two clonal P3 lines. Comparison of gene expression using only a small subset of genes from previous publicly available bulk-seq cartilage datasets was used to support phenotypic association of one clonal line with articular cartilage and the other with hypertrophic cartilage.
R1a: While the title suggests the identification of a regeneration program, the results do not adequately support this claim. I suggest either additional characterization of the regenerative process in their model or a revised title to reflect the main findings of the manuscript.
R1a response: We strongly disagree with this assessment and believe the title reflects the most important finding of the study. What the reviewer omits in the summary of findings is the demonstration that along with establishing a protocol for articular chondrocyte differentiation, a separate non-chondrogenic tissue differentiated that contained sequestered progenitor cells. The sequestration of progenitor cells during chondrogenic differentiation into a distinct tissue layer is unique and foreshadows a future regenerative need. As such, the finding identifies a regenerative characteristic that has been selected for during evolution and indicates the existence of an articular cartilage regeneration program. It is this important characteristic that distinguishes our findings from other culture strategies for the differentiation of articular chondrocytes. Progenitor cell sequestration has been demonstrated in other regenerative musculoskeletal tissues such as muscle and bone, but this is the first demonstration of progenitor cell sequestration during differentiation of a non-regenerative tissue. These results indicate the existence of an endogenous regenerative program in a non-regenerative tissue and this conclusion is of considerable importance

© 2023. Published by The Company of Biologists under the terms of the Creative Commons Attribution License (https://creativecommons.org/licenses/by/4.0/).

to the regeneration field in general. It is unfortunate that this conclusion did not resonate with Reviewer 1 as we have emphasized this conclusion throughout the revised manuscript (Abstract: lines 33-35; Introduction: lines 83-87; Discussion: lines 309-319; 325-343).
Reviewer 1 Comments for the author
R1b: In Figure 1, with the clonal selection of P3 fibroblasts, how efficient is the differentiation protocol for articular cartilage vs the parent P3 line?
R1b response: - We identified 29 clonal lines from a single 96 well plate so the efficiency was 30.2%. We have added the efficiency of our cloning protocol (line 113).
R1c: The authors should quantify the percentage of Acan+;ColX- to Acan-;ColX+ clones to see whether clonal selection and/or BMP9 treatment results in any bias towards hypertrophic or articular fate.
R1c response: We have analyzed a total of 16 chondrogenic clonal lines for expression of Acan and ColX based on immunohistochemical staining. All 16 lines were positive for Acan and 7 were negative for ColX (C6, C8, D8, D12, F1, F8, G11) whereas 9 clones were positive for ColX (C4, C9, D5, D11, E1, E3, E9, F12, G9). Thus, 44% of the clonal lines were articular and 56% were hypertrophic. This suggests that there may be a subtle selective bias in the cloning protocol for hypertrophic chondrocytes however it did not preclude the isolation of articular clonal lines. We have added the data to the manuscript (lines 125-127).
R1d: In Figure 2, the morphology of the BMP2-treated P3E3 aggregates in J has an elongated almost fibroblast-like appearance instead of spherical morphology. What can account for this changed morphology?
R1d response: We apologize for figure 2J. Unlike the other cell aggregate images (2A, 2D and 2G) where the focal plane was well above the substrate, the focal plan in figure 2J was at substrate level so cell morphology of adherent cells distracted from the aggregate. Adherent cells are present in all of the cultures and the cells appear fibroblast-like as noted by the reviewer. We have replaced figure 2J with an image taken at a focal plane above substrate level and thank the reviewer for noticing this inconsistency.
R1e: BMP2 has previously been reported to induce osteogenesis in culture; Is the BMP2 treatment inducing bone formation in P3E3 cells?
R1e response: We have tested this hypothesis by continual BMP2 treatment (18 days) of parental P3 cells, P3D8 cells and P3E6 cells and we do not observe either chondrogenesis or osteogenesis under our culture conditions. Our studies lead us to conclude that BMP2 does not induce bone formation in P3E3 cells with the caveat that unlike other studies we tested BMP2 under low serum (2%) culture conditions.
R1f: Also, the P3E3 cells are described as having a "poor self-aggregation response" (Fig 2D, line 158). Is this due to decreased proliferation of these progenitors in response to BMP2 compared to P3D8 cells?
R1f response: We have not documented cell proliferation during aggregation because it is difficult to distinguish aggregating versus non-aggregating cells in suspension culture. The poor aggregation response (Fig 2D, line 141) that the reviewer notes refer to aggregation P3E3 cells in basal medium without BMP2 and not in response to BMP2. In response to BMP2 we find robust aggregation as shown in Fig 2J.
R1g: The images in Figure 2C and 2I are very close cropped. Could the authors show images representing the full cross section of these colonies and quantify and comment on how the overall size of these colonies and their differentiation profile compare to BMP2-SA4d->BMP940d?
R1g response: Figures 2C and 2I were presented at the same magnification as Figure 2F and 2I to show the relative size difference between the differentiated aggregates, and to note the distinctive chondrogenic response of articular cartilage clones. In response to the reviewer's request, we now show the full cross section of the articular chondrogenic clusters at a lower magnification (fig 2F and 2I) and add insets to show chondrocytes and secreted matrix at a higher magnification in fig 2F (line 140) and 2I (line 154). The figure legend on Figure 2 has been appropriately modified (line 647 and line 653). With respect to the size of the chondrogenic clusters, the size of large aggregates at both time points is similar but there are distinct differences in differentiation based on histology. A comparison of the 14-day aggregate to the 40-day aggregate has been added (line 219-221).
R1h: Staining and quantification of % cells expresssing articular cartilage markers at the 14d phase (Prg4b, Cilp) should be used to show whether the additional maturation phase in culture is required for the induction of organization. Are the fibrous cells present in the colony periphery at 14d (it looks as if they are present surrounding the small cultures in Fig 2F)?
R1h response: The 14-day and the 40-day aggregates both display an organization with small chondrocytes centrally and larger chondrocytes on the periphery so the chondrocyte organization is

© 2023. Published by The Company of Biologists under the terms of the Creative Commons Attribution License (https://creativecommons.org/licenses/by/4.0/).

established by 14 days. This organization is very distinct in 40-day samples compared to the 14d samples. In our analysis we focused on the 40-day samples with histological and immunohistochemical analyses and the 14-day samples for RNAseq studies. With respect to articular cartilage related genes expressed at the 14-day timepoint: Acan is expressed in BMP9 induced cartilage (Fig 1), Cilp is differentially expressed compared to P3E3 cartilage (STable 2) and the RNAseq database indicates that Prg4 is expressed at a low level in both P3D8 and P3E3 cartilage. The fibrous external layer is present at 14 days as shown in the new low magnification images (Fig 2C and 2I). It is difficult to determine if the fibrous cells associated with the very small cartilage clusters in Fig 2F form a distinct layer. Fibroblasts are present in all samples, but the differentiation of a distinct tissue layer is unique to the P3D8 cultures. The level of cartilage maturation is not easily distinguished based solely on histology or gene expression, but we have carried out in vivo grafting of cartilage differentiated for 14, 26 and 40 days, and only the 40-day cultures maintain a chondrogenic phenotype 90 days after implantation. These results suggest that extended cultures allow cartilage maturation which is critical for the maintenance of phenotype following engraftment. We have added this observation in our description of transplantation studies (lines 276-279).

R1i: For the genome-wide analysis, it should be explicitly stated in the text and/or methods section which differentiation protocol and culture endpoint was used on the P3 lines prior to sequencing as they are marked only as P3/BMP9.

R1i response: Thank you for pointing out this confusion. The differentiation protocol and culture endpoint is stated in the text at the start of this section (line 158). Additionally, we have modified the figure 3 legend to clarify the P3D8 and P3E3 the differentiation protocol and culture endpoint used for the study (lines 658-659).

R1j: As the authors point out, previous studies have well established the similarities between the gene expression profiles of growth plate and articular cartilage, particularly with overlapping zone-specific profiles (eg. Chao 2014). Given that the two datasets the authors use for their RNAseq references come from two different studies, it is concerning that the 30 genes used to define growth plate are primarily histone and RNA genes. Is this gene signature due to the biology of the tissue or due to batch effects or other technical difference in the data? Validation in vivo or from the literature showing differential expression of these genes would assist in the interpretation of this comparison. Alternatively, would the correlation analysis using the entire gene set rather than the tope 60 DE genes or using previously published zonal laser-capture microdissection datasets from both the AC and GP (Chao 2014) provide a clearer picture of the molecular phenotype of these P3 derived chondrocytes.

R1j response: In the original manuscript, we explored different approaches to establish molecular differences between these two subpopulations of chondrocytes that are histologically distinct. The correlation analysis based on the entire gene sets from the literature indicated little differences between articular and hypertrophic cartilage (r = +0.78 where r = +1.0 indicates identity and r = -1.0 indicates the absence of identity (line 163). Next differentially expressed genes were used for the correlation analysis but this yielded r = +0.65 (line 167). By focusing only on the top 30 differentially expressed genes for each tissue we arrived at the negative correlation value, r = -0.33, and we used this gene set as a comparative reference point to analyze the cartilage differentiated by our protocol (lines 169). Reviewer 1 suggested additional approaches that we have now evaluated. First, we used the top 60 genes after removing all the histone and RNA genes but this increased the correlation value from r= -0.33 to r = -0.02. This suggests that the histone and RNA genes are important for distinguishing growth plate cartilage from articular cartilage and not due to batch effects or technical difference in the datasets. We also used articular cartilage and growth plate cartilage marker genes identified from laser-capture microdissection microarray datasets reported by Chao et al., 2014. Using the differentially expressed genes from the Chao et al. dataset we obtained a correlation of r = +0.57, again indicating that this dataset did not distinguish growth plate chondrocytes from articular chondrocytes. We note that the r = +0.57 value is similar to the value we obtained by analyzing all differentially expressed genes from the two distinct datasets (r = +0.65) suggesting that using datasets from separate studies does not bias the outcome. These analyses lead to the conclusion that the approach originally described in the manuscript provides that best resolution for distinguishing growth plate chondrocytes from articular chondrocytes based on gene expression, so we have not changed the correlation analysis in the revised manuscript.

R1k: In Figure 4C, the authors demonstrate differential gene expression in the two clonal populations. As these may be essential to driving the difference in cell phenotype or regenerative capacity, it would be helpful to either label on the volcano plot some of the key growth factor and

© 2023. Published by The Company of Biologists under the terms of the Creative Commons Attribution License (https://creativecommons.org/licenses/by/4.0/).

chondrogenic genes discussed in the text and/or to include the fold change information for these key genes to illustrate how the expression compares between the two clonal lines. This would aid the interpretation of the significance of the discussed genes and provide more mechanistic insights into the differentiation phenotypes of the two clonal lines. In addition to more details regarding the few chondrocyte genes listed, a more in-depth characterization of these genetic differences to show the global genetic changes in gene set enrichment (eg. TFs, growth factor signaling pathway activity) between the different differentiation trajectories could be used to more clearly define the regeneration program suggested by the title.
R1k response: Thank you for this suggestion. We have modified the volcano plot to identify the most highly up regulated genes (see figure 3C). We have also added a paragraph describing the identification of transcription factors that are up regulated in the two cartilage types, and the results of a KEGG pathway enrichment analysis that shows global genetic changes in the datasets (lines 199-213).
R1l: line 275, the authors state that the P3D8-F1 isolated cells and P3D8 were similarly proliferative. Can the authors please provide these quantitative data.
R1l response: We do not have quantitative data. This statement was based on the similarity in time to reach confluency for P3D8/F1 cells compared to the P3D8 cells. We have modified the statement for accuracy (line 256).
R1m: The implant data in Figure 6 nicely shows persistent Safranin-O/Sox9+ graft survival after a 90-day period. The authors state on line 319 that these implants retain articular cartilage characteristics. To support defining the articular cartilage phenotype, it would be important to show whether the implants retain Prg4b and Cilp expression.
R1m response: Reviewer 1 makes a good point. In the original manuscript we were able to demonstrate engrafted tissue expression of the cell marker (ß-gal), and marker proteins Acan, Sox9, ColX and ColI in adjacent sections so we were confident that the expression or lack of expression was linked to engrafted tissue. In response to the referee's suggestion, we developed double immunostaining for ß-gal with Prg4 and Cilp to explore this question in another group of engrafted tissue samples. The results show that engrafted cells do not co-express ß-gal and Prg4 or ß-gal and Cilp indicating engrafted cells do not retain expression of all articular markers demonstrated in vitro. We have added co-immunostaining images to Figure 6 (Fig 6I and 6J), describe these negative findings (lines 292-294), and have modified our conclusion to indicate that not all articular cartilage markers are retained after transplantation (line 308).
R1n: In general, the authors should include n numbers for all culture and staining experiments.
R1n response: We have included n numbers in both the text and figure legends for all experiments. Thank you for pointing this out.

Reviewer 2 Advance summary and potential significance to field
Lin and colleagues have refined a cell culture organoid model using fibroblasts from the terminal phalanx (P3) of mice, to generate articular cartilage through sequential treatment with BMP2 and BMP9. This work appears to be a follow-on study from their previous publication whereby the use of digit tip fibroblasts or their ability to generate articular cartilage has been previously described (Yu et al, 2022, Development). The authors however, have presented three new advances; (a) refinement of the culturing protocol which involves sequential treatment of fibroblasts with BMP2 and BMP9 leading to enhanced aggregation of 'chondro-spheres'; (b) articular cartilage self-organises in culture generating a layer of encapsulating fibrous connective tissue; and (c) the transplantation of the newly generated articular cartilage was stable in vivo. These findings represent an advancement to our current knowledge and would be of broad interest to developmental biologists and the regenerative medicine communities. The use of digit tip fibroblasts to generate cartilage is a novel and understudied area of research. Furthermore the study provides methodological advances in the generation of articular cartilage and characterisation of its potential for use in therapeutic applications. Since cartilage does not endogenously regenerate in mammals, its damage leads to impaired joint function and represents a significant burden on the health care system. Overall, I am supportive of the work described in this manuscript, however, would suggest some revisions as outlined below.

Reviewer 2 Comments for the author
R2a: The authors use fibroblasts derived from the distal phalanx (P3) of the mouse digit tip. This could contain cells from the periosteum which contain a resident source of osteochondro-progenitors. Can the authors be certain they are not just amplifying clonal lines of these specific

© 2023. Published by The Company of Biologists under the terms of the Creative Commons Attribution License (https://creativecommons.org/licenses/by/4.0/).

osteochondroprogenitors rather than fibroblasts having the potential to generate chondrocytes? cultured in MSC media for a prolonged period.
R2a response: The question of whether P3 cells are MSC or SSC was addressed in our previous paper (PMID: 35005773) where we show that P3 cells are chondrogenic and adipogenic and not osteogenic based on traditional differentiation assays. The question of whether P3 cells are fibroblasts was also addressed in part in our previous study. We began studying P3 cells because of their similarity to wound cells derived from non-regenerative P2 amputations (P2 amputation wound cells) that are chondrogenic in response to BMP9 in vivo (PMID: 30723209) and in vitro (PMID: 35005773). P2 amputation wound cells were shown to be fibroblastic based on scRNAseq transcriptomic data (PMID: 35005773). In addition to published data, as part of ongoing studies, we have unpublished scRNAseq datasets for P3 cells and P3D8 clonal cells. P3 cells and P2 amputation wound cells express a similar array of key fibroblast marker genes (PMID: 33981032) and as expected, P3D8 cells express a subset of these genes, indicating their fibroblastic nature. tSNE mapping of P3 and P3D8 cells display overlap with each other but no overlap with published datasets for MSCs or SSCs. Based on published and unpublished data, we are confident that P3D8 cells represent a unique fibroblast chondroprogenitor cell and not a clonal line of established osteochondroprogenitors.
R2b: Do you obtain similar results from cells taken directly from P3 digits?
R2b response: The answer is yes, we do obtain similar results from cell taken directly from P3 digits. This was reported in our previous paper (PMID: 35005773) along with results that are comparable to the P3 cell line used for cloning. The procedure is simple and highly reproducible, and we have generated P3 cells from a number of different strains, different age mice, and mouse lines used to study genetic anomalies (e.g. Downs Syndrome).
R2c: Can the authors comment on why their protocols to generate articular cartilage only correlates to in vivo articular cartilage with a p = 0.51? This is a relatively low correlation for similar cell types. A trajectory analyses using Monocle or RNA velocity might be more convincing here to show they are on their way to becoming articular cartilage (this would however require single cell RNA sequencing data rather than the bulk RNA sequencing data provided in the paper).
R2c response: We agree that this is a relatively low correlation, but it is important to understand that the articular cartilage dataset we are using as a reference is composed of both articular and hypertrophic chondrocytes, whereas the cartilage differentiated in culture is either articular or hypertrophic. We have added clarification in the revised manuscript (lines 180-183). In addition, the articular cartilage differentiated from P3D8 cells also consist of the fibrous encapsulating layer that is non-chondrogenic and absent in joint articular cartilage preparations. It is important to keep in mind that the correlation analysis is used to tease out differences between growth plate cartilage and articular cartilage which are very similar when the entire datasets are compared (r = +0.78) and even when only differentially expressed genes are compared (r = +0.65). By restricting the analysis to the top differentially expressed genes we were able to detect a difference (r = -0.33) that could be used to explore P3D8 and P3E3 induced cartilage. We conclude that the correlation of r = +0.5 represents a relative trend toward articular cartilage and not growth plate cartilage. While this result would not stand on its own, in combination with histological and immunohistochemical evidence the data support the conclusion that both P3D8 and P3E3 derived cartilage are articular.
R2d: Line 226 – The authors state that organized articular cartilage differentiates from P3D8 clonal cell cultures that has a structurally firm tissue that was resilient to pressure. How was this measured?
R2d response: This was not quantitative but an observation from cutting and handling the differentiated articular cartilage during transplantation. We have moved this observation to the section of Results that describe transplantation of cartilage into injured digit joints (lines 276-279).
R2e: Following transplantation of articular cartilage into the acute joint defect, there appears to be an abundance of Sox9 positive progenitor cells. This did not seem to be the case in vitro. Can the authors explain why Sox9 expression has increased?
R2e response: This is an interesting observation that we had not appreciated. Almost all engrafted chondrocytes express Sox9 (Fig 6D whereas Sox9 is expressed in about 50% of the chondrocytes in vitro (Fig 4H). Sox9 is expressed in most cells of the knee articular cartilage (SFig 1C) so the engrafted cells are most similar to the in vivo condition. We do not have a good explanation for this but selection for Sox9 positive chondrocytes may enhance engraftment success. We have added this observation to the text (lines 100, 235, 289) and thank the reviewer for bringing this to our attention.

© 2023. Published by The Company of Biologists under the terms of the Creative Commons Attribution License (https://creativecommons.org/licenses/by/4.0/).

R2f: Was the transplanted articular cartilage functional? (i.e. Did the mice gain back normal gait for example?)
R2f response: We have analyzed gait following digit joint injury but unfortunately mouse gait is not modified by this joint injury. We are still searching for a functional assay for the studies.

Additional Minor Comments

R2g: The authors use phrases (line 75) such as 'The chondrogenic response of P3 fibroblasts was explored by cloning cells and characterizing responsiveness to BMP9'. The use of the word cloning here is ambiguous (generally a molecular biology term). It would be better to state they generated clonal cell lines. This is also the case in the abstract.
R2g response: This has been changed (lines 28, 75).
Reviewer 3 Advance summary and potential significance to field
This manuscript describes a new organoid model to study the induction of cartilage tissue using BMP2+9. Several clonal mesenchymal cell lines could induce cartilage with different genetic signatures and alternative differentiation potentials. Induced cartilage could spontaneously form stratified layers of chondrocytes that could be implanted into surgical joint defects in vivo with retention of overall structure and immunoreactivity for several chondrocyte marker genes. This work represents an exciting step forward in understanding the potential for regeneration in mammalian tissues. The finding that chondroprogenitor cells are sequestered into the fibrous connective tissue layer that spontaneously forms in the BMP2+9 induction protocol is extremely interesting and reveals potential parallels with other regenerative contexts giving weight to the idea that mammals have more regenerative potential than previously thought. Overall, the manuscript is well written and represents a good resource for the field.

Reviewer 3 Comments for the author
Below are some comments that are designed to improve clarity in the manuscript.
R3a: 1)In supplemental figure 1. The authors could improve clarity by indicating exactly what the dotted line represents in D-F. Prg4+ chondrocytes butted up at the edge of the superficial zone. Check the placement of the dotted line in E as Clip+ Chondrocytes also appear to be placed at boundary of superficial zone and middle zone.
R3a response: We have included in supplemental figure 1 legend that the dotted lines identify the boundary of the articular cartilage surface abutting the joint cavity. The joint cavity is compressed in this section, so the two abutting surfaces are in contact. The placement of the dotted line in E is correct and we find some Cilp+ cells in the superficial zone at this stage of development. We have as added this to the text (line 103).
R3b: 2)The statement made on line 234 seems a little confusing when figure 4 is compared with supplemental figure 1. When one compares the marker gene localization with the developing postnatal neonatal knee cartilage the statement seems to be inconsistent. The authors state that: "Overall, the BMP2-SA4d->BMP940d differentiated P3D8 cartilage was organized into concentric rings of chondrocytes displaying a gradient of increasing chondrocyte size and glycoaminoglycan production that parallels the middle and deep zones of maturing articular cartilage (Supplemental Fig 1A). Immunostaining studies also supported this conclusion;"
However, in the p7 developing knee cartilage (Supp figure 1) they show:
C) Cells of the joint forming region display uniform expression of Sox9.
D) Chondrocytes of the superficial zone (arrow) are small and express Prg4.
E) Middle zone chondrocytes (arrow) are small with lacunae and express Cilp.
F) Chondrocytes of the deep zones (arrow) have large lacunae and express Acan.
G) Columns of hypertrophic chondrocytes of the growth plate (arrow) express ColX.
H) Surrounding fibrous connective tissue (arrow) are non-chondrogenic and are positive for ColI expression.
In organized articular cartilage differentiates from P3D8 clonal cell cultures they report:
-Sox9 expressing cells were not localized and abundant throughout the regenerated cartilage (Fig 4H)
-Prg4 expressing cells were found scattered throughout the cartilage and was not localized (Fig 4K; Supplemental Fig 3D).
-Cilp immune-positive cells were abundant in the central core of small chondrocytes while
-Acan immune-positive cells were most abundant in the peripheral region of larger chondrocytes (Fig 4F,G; 238 Supplemental Fig 3A-C).
-while ColX and ColI immune-positive cells are absent in the differentiated cartilage (Fig 4I,J).

© 2023. Published by The Company of Biologists under the terms of the Creative Commons Attribution License (https://creativecommons.org/licenses/by/4.0/).

It would be extremely helpful for the reader if the authors could write something to describe the contrast in these patterns. It is probably not unreasonable that the patterns would be different considering the extrinsic signals (or lack thereof) in the artificial culturing microenvironment, but these differences should be acknowledged and discussed.
R3b response: Thank you for pointing out this confusion. We have modified the description and comparison to in vitro regenerated cartilage to highlight similarities as well as differences articular cartilage in situ (lines 226-238 and 383-387).
R3c: Despite this pattern mismatch, it is interesting that regenerated articular chondrocytes are organized into concentric layers (possibly inverted?) and that further studies on extrinsic factors could provide important insights into this differentiation and organization.
R3c response: We agree. This is discussed on lines 387-394.
Minor comments:
R3d: 3)There may be a missing word on line 63. "Chondrocytes of the superficial zone are small, flattened 63 cells containing relatively low proteoglycan levels and are identified based on proteoglycan 4 (Prg4, also called lubricin) expression of (Saito, 2022)."
R3d response: We have corrected the typo (line 65).
R3e: 4)In the discussion lines 395-398., they self-aggregate and differentiate hyaline cartilage when induced with BMP9 (Yu et al., 2022). Is this supposed to read BMP2? (.BMP2 is supposed to induces the self-aggregation and BMP9 induces the differentiation, right?)
R3e response: The text refers to the parental P3 cell line and not the clonal lines. The parental P3 cell line readily aggregates in suspension and the protocol in the Yu et al., 2022 study was to aggregate cells without growth factor treatment then to differentiate the aggregates with BMP9. We have added "digit derived P3 fibroblast" to the sentence to make it clear we are not referring to clonal cells (Line 360).
R3f: 5)Supplementary table I seems to be missing from the manuscript PDF.
R3f response: Supplemental Table 1 is an excel file that was uploaded with the manuscript.

Second decision letter

MS ID#: DEVELOP/2023/201894

MS TITLE: Induced regeneration of articular cartilage: Identification of a dormant regeneration program for a non-regenerative tissue

AUTHORS: Yu-Lieh Lin, Ling Yu, Mingquan Yan, Katherine Zimmel, Osama Qureshi, Felisha Imholt, Tao Li, Ivan Ivanov, Regina Brunauer, Lindsay A Dawson, and Ken Muneoka
ARTICLE TYPE: Research Article

I am happy to tell you that your manuscript has been accepted for publication in Development, pending our standard ethics checks. Please consider revisions to the title as suggested by Reviewer #3.

Reviewer 1

Advance summary and potential significance to field

The generation of articular cartilage for engraftment is a highly significant problem in regenerative biology. Identifying the optimal cell source for ex vivo articular cartilage generation is a key goal in the field. Previously, this group identified that P3 digit fibroblasts are a cell population that can be grown in the presence of BMP9 in organoid culture to resemble articular cartilage and survive engraftment into joint in vivo. The present study involves clonal selection of P3 fibroblasts and the refinement of culture protocols (BMP2->BMP9) using one of these clonal lines to promote articular over hypertrophic cartilage fate in self-aggregating organoid culture. In their novel protocol, they find expression of key markers of articular cartilage in these fibroblast-derived organoids. Finally they show that in vitro organoid-derived chondrocytes can survive engraftment in vivo and maintain expression of some articular cartilage markers. These studies will be of interest not only to the cartilage biology community but also within the greater regenerative biology field.

© 2023. Published by The Company of Biologists under the terms of the Creative Commons Attribution License (https://creativecommons.org/licenses/by/4.0/).

Comments for the author

We thank the authors for thoughtfully and thoroughly addressing our suggested revisions.

Reviewer 2

Advance summary and potential significance to field

Lin and colleagues have refined a cell culture organoid model using fibroblasts from the terminal phalanx (P3) of mice, to generate articular cartilage through sequential treatment with BMP2 and BMP9. This work appears to be a follow on study from their previous publication whereby the use of digit tip fibroblasts or their ability to generate articular cartilage has been previously described (Yu et al, 2022, Development). The authors however, have presented three new advances; (a) refinement of the culturing protocol which involves sequential treatment of fibroblasts with BMP2 and BMP9 leading to enhanced aggregation of ‘chondro-spheres’; (b) articular cartilage self-organises in culture generating a layer of encapsulating fibrous connective tissue; and (c) the transplantation of the newly generated articular cartilage was stable in vivo.
These findings represent an advancement to our current knowledge and would be of broad interest to developmental biologists and the regenerative medicine communities. The use of digit tip fibroblasts to generate cartilage is a novel and understudied area of research. Furthermore the study provides methodological advances in the generation of articular cartilage and characterisation of its potential for use in therapeutic applications. Since cartilage does not endogenously regenerate in mammals, its damage leads to impaired joint function and represents a significant burden on the health care system.

With the current revisions to the manuscript, I am supportive of its publication without further modification.

Comments for the author

I am satisfied that the authors have made reasonable revisions to the manuscript that satisfy my suggestions/comments and recommend acceptance of the article.

Reviewer 3

Advance summary and potential significance to field

This work advances our understanding of articular cartilage induction and paves the way for future organoid/cell transplantation approaches and regenerative therapies.

Comments for the author

The authors have made significant improvements to the original manuscript, and I fully endorse the publication of this exciting work. One of the main findings of interest in this paper although Articular cartilage is not normally regenerative a protocol of AC progenitor induction can be used in vitro to form AC organoid with an organization that sequesters progenitor cells within a layer of fibrous connective tissue and that some characteristics (both physical and immunohistochemical) are maintained after transplantation in vivo (although some are lost). This has obvious implications for future cell/organoid transplant therapies but the detailed molecular events surrounding this phenomenon are still a little vague. As such, although the title does link to the major findings the exact wording of the title does imply that the paper will describe an in vivo molecular process that will detail the dormant regenerative program.
Once could consider a slight modification of the title to more accurately reflect the data?
“Induced regeneration of articular cartilage organoids: Identification of dormant regenerative characteristics in a non-regenerative tissue, partially maintained post-transplantation.”

© 2023. Published by The Company of Biologists under the terms of the Creative Commons Attribution License (https://creativecommons.org/licenses/by/4.0/).
